# *In silico* design of novel bioactive molecules to treat breast cancer with chlorogenic acid derivatives: a computational and SAR approach

**DOI:** 10.3389/fphar.2023.1266833

**Published:** 2023-12-12

**Authors:** Renu Sehrawat, Priyanka Rathee, Pooja Rathee, Sarita Khatkar, Esra Küpeli Akkol, Anurag Khatkar, Eduardo Sobarzo-Sánchez

**Affiliations:** ^1^ School of Medical and Allied Sciences, K. R. Mangalam University, Gurugram, Haryana, India; ^2^ Faculty of Pharmaceutical Sciences, Baba Mastnath University, Rohtak, India; ^3^ Department of Pharmaceutical Sciences, Maharshi Dayanand University, Rohtak, Haryana, India; ^4^ Vaish Institute of Pharmaceutical Education and Research, Rohtak, Haryana, India; ^5^ Department of Pharmacognosy, Faculty of Pharmacy, Gazi University, Ankara, Türkiye; ^6^ Instituto de Investigación y Postgrado, Facultad de Medicina y Ciencias de la Salud, Universidad Central de Chile, Santiago, Chile; ^7^ Department of Organic Chemistry, Faculty of Pharmacy, University of Santiago de Compostela, Santiago de Compostela, Spain

**Keywords:** molecular docking, chlorogenic acid, breast cancer, drug development, pharmacokinetic, *In silico* design

## Abstract

**Introduction:** Cancer is a vast group of diseases comprising abnormal cells that multiply and grow uncontrollably, and it is one of the top causes of death globally. Several types of cancers are diagnosed, but the incidence of breast cancer, especially in postmenopausal women, is increasing daily. Chemotherapeutic agents used to treat cancer are generally associated with severe side effects on host cells, which has led to a search for safe and potential alternatives. Therefore, the present research has been conducted to find novel bioactive molecules to treat breast cancer with chlorogenic acid and its derivatives. Chlorogenic acid was selected because of its known activity in the field.

**Methods:** Several chlorogenic acid derivatives were subjected to computational studies such as molecular docking, determination of absorption, distribution, metabolism, and excretion (ADME), druglikeness, toxicity, and prediction of activity spectra for substances (PASS) to develop a potential inhibitor of breast cancer. The Protein Data Bank (PDB) IDs used for docking purposes were 7KCD, 3ERT, 6CHZ, 3HB5, and 1U72.

**Result:** Exhaustive analysis of results has been conducted by considering various parameters, like docking score, binding energy, types of interaction with important amino acid residues in the binding pocket, ADME, and toxicity data of compounds. Among all the selected derivatives, CgE18, CgE11, CgAm13, CgE16, and CgE9 have astonishing interactions, excellent binding energy, and better stability in the active site of targeted proteins. The docking scores of compound CgE18 were −11.63 kcal/mol, −14.15 kcal/mol, and −12.90 kcal/mol against breast cancer PDB IDs 7KCD, 3HB5, and 1U72, respectively. The docking scores of compound CgE11 were −10.77 kcal/mol and −9.11 kcal/mol against breast cancer PDB IDs 3ERT and 6CHZ, respectively, whereas the docking scores of epirubicin hydrochloride were −3.85 kcal/mol, −6.4 kcal/mol, −8.76 kcal/mol, and −10.5 kcal/mol against PDB IDs 7KCD, 3ERT, 6CHZ, and 3HB5. The docking scores of 5-fluorouracil were found to be −5.25 kcal/mol, −3.43 kcal/mol, −3.73 kcal/mol, and −5.29 kcal/mol against PDB IDs 7KCD, 3ERT, 6CHZ, and 3HB5, which indicates the designed compounds have a better docking score than some standard drugs.

**Conclusion:** Taking into account the results of molecular docking, drug likeness analysis, absorption, distribution, metabolism, excretion, and toxicity (ADMET) evaluation, and PASS, it can be concluded that chlorogenic acid derivatives hold promise as potent inhibitors for the treatment of breast cancer.

## 1 Introduction

Cancer is a colossal group of diseases that can start in any part of the body and then invade adjacent or other body parts when abnormal cells multiply and grow uncontrollably (Van Vo et al., 2022). It is one of the top causes of death globally, and the incidence rate is increasing day by day. It is one of the primary contributors to global mortality, with its incidence steadily rising. According to a report from the World Health Organization, cancer is the leading cause of death, accounting for nearly 10 million deaths in 2020, or nearly one out of every six deaths (World Health Organization, 2018). Many cancer types are identified, with the most prevalent being breast, lung, brain, colon, blood, prostate, stomach, and liver cancers. However, it is worth noting that breast, lung, and thyroid cancers are more frequently diagnosed in women (Akash, 2021; Rahib et al., 2014). In 2020, the most prevalent cancer diagnoses included breast cancer (2.26 million cases), lung cancer (2.21 million cases), colon and rectal cancer (1.93 million cases), prostate cancer (1.41 million cases), non-melanoma skin cancer (1.20 million cases), and stomach cancer (1.09 million cases). Among these, breast cancer exhibited a stronger association with the female gender, resulting in 685,000 global deaths in 2020. Women between the ages of 40 and 60 exhibited a higher susceptibility to breast cancer, accounting for approximately 75% of all cases. In contrast, the female population under the age of 30 had a mere 5% chance of developing breast cancer, while those aged 60 and above constituted 20% of breast cancer cases. This report underscores that the age group of 40–60 is associated with the highest incidence of breast cancer (Küpeli Akkol et al., 2022; Majid et al., 2017).

A breast is made up of three major parts: connective tissue, ducts, and lobules, and breast cancer can start from any part (Centers for Disease Control and Prevention, 2022). In most cases, it initially arises in the epithelium cells of the ducts (85%) or lobules (15%) in the glandular tissue of the breast, where generally no symptoms are observed (no metastasis). Over time, it spreads to nearby lymph nodes (regional metastasis) or other body parts (distant metastasis). Several types of breast cancer depend on the specific type of breast cells affected (Łukasiewicz et al., 2021; Neve et al., 2006; Bender and Atalay, 2018; American Cancer Society, 2022).

Chemotherapy, surgery, and radiotherapy are mainly used to treat cancer, but they are associated with severe side effects on host cells, which has led to a search for new alternatives (Newman and Cragg, 2016). To overcome the limitations of current chemotherapeutics, researchers turned their attention toward developing natural compounds as chemotherapeutic agents (Küpeli Akkol et al., 2020b; Cragg and Pezzuto, 2016; Sehrawat et al., 2022). While many anticancer drugs have originated from natural sources, it is important to acknowledge that many plant compounds with anticancer potential remain largely unexplored in drug discovery. Despite the successes achieved with natural anticancer compounds, numerous plant constituents have yet to be systematically investigated for their therapeutic potential. Phytochemicals could be used as alternatives to synthetic chemotherapeutic agents as they have anticancer activities and can protect vital cellular components like DNA, proteins, and lipids against oxidation (Küpeli Akkol et al., 2020a; Saibabu et al., 2015; Cragg and Newman, 2005).

Chlorogenic acid (CGA) is a phenolic acid composed of caffeic acid and quinic acid linked by an ester bond. Chlorogenic acid has vast biological applications due to its antioxidant, antiviral, antidiabetic, anti-inflammatory, antimicrobial, and anticancer activity (Johnston et al., 2003; Kaur et al., 2022; Schuster et al., 2022; Yang et al., 2022; Wang et al., 2020; Yan et al., 2017; Jassim and Naji, 2003; Clifford, 2000; Morton et al., 2000). Many studies have reported that chlorogenic acid has immense antitumor activity against breast cancer by inhibiting NF-κB, inhibiting the β-catenin of the Wnt signaling pathway, and also inhibiting proliferation, viability, and suppressing invasion and migration of breast cancer cells (Zeng et al., 2021; Bender and Atalay, 2021). Hayakawa et al. (2020), Zeng et al. (2021), Murai and Matsuda (2023), and Gupta et al. (2022) have all identified chlorogenic acid (CGA) as a promising candidate for anticancer therapy. Nwafor et al. (2022) reported that CGA could be a valuable treatment resource for breast cancer because it inhibits macrophage M2 polarization. CGA also induces apoptosis, impedes metastasis, and enhances antitumor immunity via the NF-κB signaling pathway. Although CGA has been relatively underexplored in previous studies, there are limited reports available in the literature on its derivatization. The lack of research into CGA derivatives highlights the unexplored potential for the discovery of novel compounds with a diverse range of pharmacological effects. Notable studies include chlorogenic acid-based peptidomimetics by Daneshtalab (2008), which introduced a new class of antifungal agents. Pressete et al. (2023) explored the use of a piperine–chlorogenic acid hybrid for treating skin cancer, and Kataria and Khatkar (2019) investigated chlorogenic derivatives for their possible use as urease inhibitors.

Therefore, CGA appears to be the most promising lead for chemical alteration for the development of novel and effective chemotherapeutics. To the best of our knowledge, no extensive computational investigation has been performed to discover novel and safe chlorogenic acid derivatives as chemotherapeutic agents against breast cancer with improved pharmacokinetic properties. Keeping this in mind, the study was performed to identify the pharmacophore required to interact with essential amino acid residues. The design and development of a novel drug is a tedious, costly, and time-consuming process, but these issues could be settled to some extent by the advancement in computer-based drug design, especially structural-based drug design (SBDD, or molecular docking) techniques (Rahman et al., 2012). SBDD can be used to screen thousands of ligands and predict their affinity toward a particular disease target (Lather et al., 2018). Therefore, virtual screening is one of the most widely accepted and used techniques to extract the hits and remove the non-complementary compounds (Katsila et al., 2016; Sharma et al., 2018).

In this present investigational study, we have focused on the development of novel and selective chlorogenic acid derivatives as anticancer agents using structure-based virtual screening. High-binding-score ligands were chosen and finally ascertained by calculating their absorption, distribution, metabolism, excretion, and toxicity (ADMET) properties utilizing the QikProp module. This study provides new insights and baselines for the development of a novel drug candidate for breast cancer with improved efficacy and fewer side effects. In this research, we suggested chlorogenic acid and its derivatives for the discovery of novel potential medications for the treatment of breast cancer.

## 2 Materials and methods

Docking analysis of chlorogenic acid and its designed derivatives was evaluated using Maestro Schrödinger Glide (New York, United States) software. The pharmacokinetic parameters were calculated using the QikProp tool. An Intel^®^ Core™ i5-4210U CPU @ 2.40 GHz, RAM 4.0 GB under 64-bit Windows OS was the hardware configuration. To check physiological and biological properties online, the Prediction of Activity Spectra of Substances (PASS) tool was utilized. Chlorogenic acid is readily available commercially to facilitate the advancement of this research. All *in silico* work was accomplished in the Laboratory for Enzyme Inhibition Studies, M.D. University, Rohtak, India.

### 2.1 PASS

The PASS online prediction tool was utilized to predict the biological activity spectrum of designed derivatives. The PASS computer system predicts over 3,500 kinds of biological activity, including pharmacological effects, mechanisms of action, toxic and adverse effects, interaction with metabolic enzymes and transporters, and influence on gene expression (PASS, 2022). It is represented by the Pa and Pi values.

### 2.2 Ligand preparation and optimization

ChemDraw Ultra 8.0 software was used to draw the structures of chlorogenic acids and their derivatives and save them in MDL Molfile format. The LigPrep tool of Maestro Molecular Modeling software was used to correct the coordinates and stereochemistry, generate tautomers, and minimize energy to obtain the appropriate ligand conformation. The default option was set at 32 stereoisomers per ligand at target pH 7±2, the force field was OPLS3e, and Epik was used for ionization. Ligand geometry was minimized by the application of the OPLS3e force field algorithm. Then, these energy-minimized prepared ligands were used for molecular docking simulation.

### 2.3 ADME and druglikeness study

A druglikeness calculation is very important in the drug discovery process, and the QikProp graphical interface of the Maestro Schrödinger molecular modeling suite was utilized for this purpose. The drug’s likeness is calculated by applying Lipinski’s rule of five (H-bond donors should not be more than 5 and H-bond acceptors should not be more than 10, rotatable hydrogen bonds should not be more than 10, molecular weight should not be greater than 500, and calculated Log P (CLog P) should not be greater than 5) (Benet et al., 2016). Descriptors calculated by using this module were: absorption, distribution, metabolism, Predicted brain/blood partition coefficient (QPlogBB), Predicted IC_50_ value for blockage of HERG K^+^ channels (QPlog HERG), Predicted human serum albumin binding (QPlogKhsa), permeation through skin estimation (QPlogKp), apparent Caco-2 cell permeability estimation in nm/sec (QPPCaco), apparent Madin–Darby canine kidney (MDCK) cell permeability estimation in nm/sec (QPPMDCK), Predicted partition coefficient in octanol and water (QPlogPo/w), solubility in aqueous media (QPlogS), percent human oral absorption (% HOA), and Lipinski’s rule of five and rule of three (Lipinski et al., 2012; Veber et al., 2002; Lipinski et al., 2001; Irvine et al., 1998; Kulkarni et al., 2002).

### 2.4 Protein preparation

Selected breast cancer proteins were retrieved from the Research Collaboratory for Structural Bioinformatics (RCSB) Protein Data Bank with PDB IDs: 7KCD, 3ERT, 6CHZ, 3HB5, and 1U72 (RCSB, 2022; Singh et al., 2018; Sahayarayan et al., 2021). PDB IDs were selected based on resolution and source species. Imported proteins are not directly suitable for molecular docking as they consist of heavy metals, co-crystalized ligands, water molecules, cofactors, and metal ions. Hence, protein preparation was done with the help of the Schrödinger Protein Preparation Wizard module (Schrodinger Maestro, 2020; Halgren et al., 2004; Friesner et al., 2004; Friesner et al., 2006). The targeted protein structure was further refined to obtain an optimized, chemically accurate, and energy-minimized protein structure. Proteins are directly downloaded from the Protein Data Bank on the Maestro workspace interface, followed by pre-processing steps that include assigning bond order, adding hydrogen, creating zero-order bonds to metals, converting selenomethionines to methionines, creating disulfide bonds, filling in missing side chains, and filling in missing loop chains using Prime. All the water molecules were removed, and the Epik tool was used to ionize heteroatoms at biological pH to maintain a biosimilar environment. After pre-processing, the energy-minimized structure was obtained using the OPLS3e force field. To validate the docking protocol, Root Mean Square Deviation (RMSD) values were determined and found to be below 2 Å, which is sufficient to approve the docking protocol. The crystal structures of proteins and their related information are given in Table 1.

**TABLE 1:** Breast cancer protein three-dimensional structures by PDB ID.

Protein PDB ID	Organism	Resolution (Å)	Three-dimensional structure of breast cancer protein	Reference
7KCD	*Homo sapiens*	1.80	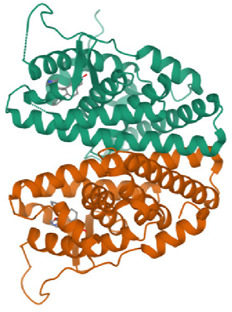	Hosfield et al. (2022)
3ERT	*Homo sapiens*	1.9	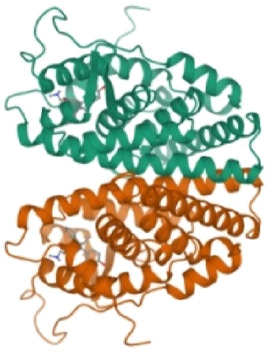	Shiau et al. (1998)
6CHZ	*Homo sapiens*	1.68	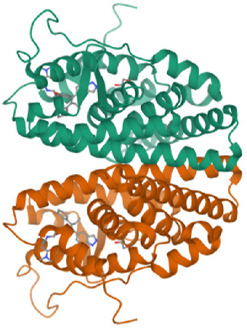	Puyang et al. (2018)
3HB5	*Homo sapiens*	2.00	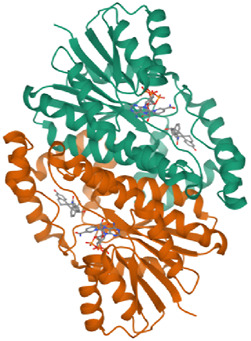	Mazumdar et al. (2009)
1U72	*Homo sapiens*	1.90	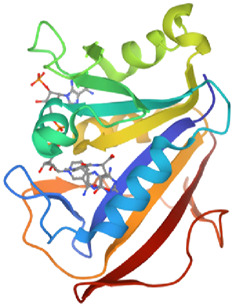	Cody et al. (2005)

### 2.5 Generation of the grid

The Maestro receptor grid generation tool was used to calculate the grids required for docking ligands to the protein receptor. A grid was generated by picking molecules from selected PDB IDs, and a docking receptor grid generation file was utilized to bind ligands to the binding site of the prepared protein.

### 2.6 Molecular docking

The Maestro XP (extra precision) Glide algorithm mode was utilized to check the interaction of ligands with prepared proteins. In the ligand docking panel, a grid file with a zip file extension was utilized to specify the binding site. To validate the docking site, RMSD was calculated by using the superposition tool of the structure alignment task of Maestro for each protein, and it was found to be less than 2 Å (García-Godoy et al., 2016). It is considered satisfactory to approve the docking protocol. The docking result was in the form of a grid-based ligand docking with energetics (glide) score.

## 3 Results and discussion

### 3.1 Structure–activity relationship (SAR)

**FIGURE 1:**
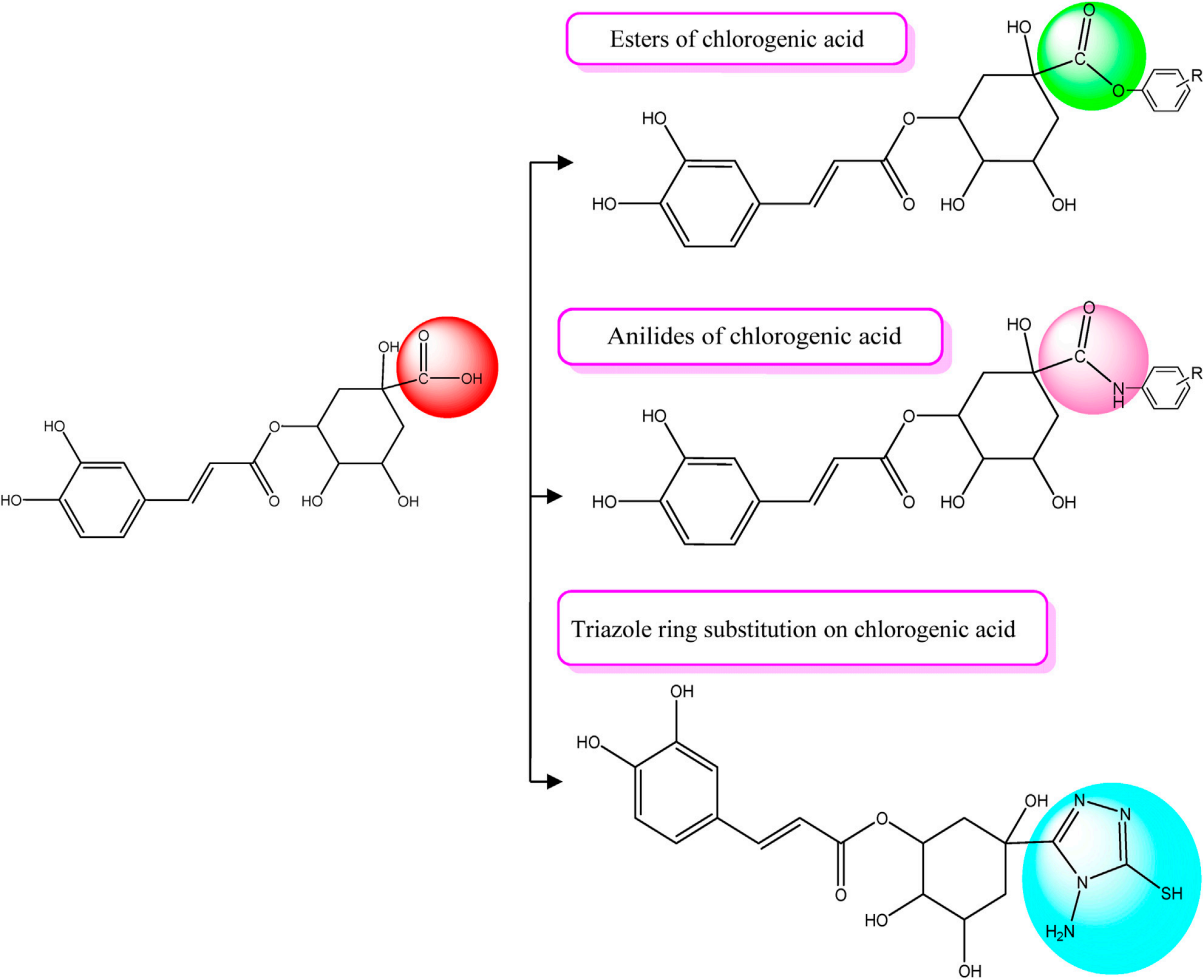
Chlorogenic acid derivative design strategies.

In previous study results (Sehrawat et al., 2023), chlorogenic acid was found to be an effective anticancer compound. Chlorogenic acid had an excellent binding score and was the most promising lead for future chemical alteration. This compound was corroborated as a lead or could be explored as a chemical template for the successive development and design of novel derivatives of chlorogenic acid with improved pharmacological activity. Chlorogenic acid possesses three distinct functional groups that can be strategically altered to modify its lead structure. These groups include the carboxylic acid, hydroxyl, and ester groups. In this context, the carboxylic acid functional group within the quinic acid ring of chlorogenic acid was specifically chosen for modification. This choice was made because the free hydroxyl groups are engaged in hydrogen bonding interactions with essential amino acid residues of the targeted proteins associated with breast cancer, as depicted in Figures 3–7. Additionally, the incorporation of ester groups was deemed beneficial for enhancing biological activity, as reported by Sánchez-del-Campo et al. (2009). The carboxylic acid group was transformed into esters, anilides, amides, and a triazole ring, all of which were integrated to augment the compound’s biological effectiveness (Figure 1). In order to investigate the structural feature relationships between newly designed chlorogenic derivatives and their biological efficacy, a SAR analysis was conducted, yielding the following observations:

• Ester derivatives outperformed anilides: Ester derivatives exhibited significantly stronger binding affinity toward the targeted protein than anilides. Notable examples of high-binding ester derivatives include compounds CgE18, CgE11, and CgE16.
• Aromatic esters showed enhanced binding: Among ester derivatives, aromatic esters displayed superior binding compared to aliphatic esters. For instance, compound CgE18 exhibited excellent binding, surpassing compound CgE9.
• Bulkier aromatic groups enhanced hydrophobic interactions: The introduction of bulkier aromatic groups in certain derivatives led to intensified hydrophobic interactions with hydrophobic amino acid residues within the breast cancer protein.
• Electron-withdrawing substitutions improved activity: Derivatives with electron-withdrawing substitutions, such as hydroxyl and methoxy groups, demonstrated increased activity. Notable examples include CgE18 and CgE16.
• Triazole ring introduction: The incorporation of a triazole ring did not result in significantly stronger binding than was observed with ester derivatives of the compound.
• High binding scores compared to standard drugs: Most of the designed compounds exhibited better binding scores against targeted proteins than standard drugs like epirubicin hydrochloride and 5-fluorouracil, with the exception of methotrexate.

These findings underscore the potential of specific chlorogenic acid derivatives, particularly those with ester substitutions, as promising candidates for further investigation in breast cancer treatment due to their strong binding affinity to the target protein.

### 3.2 Optimized structure of the tested ligand

**FIGURE 2:**
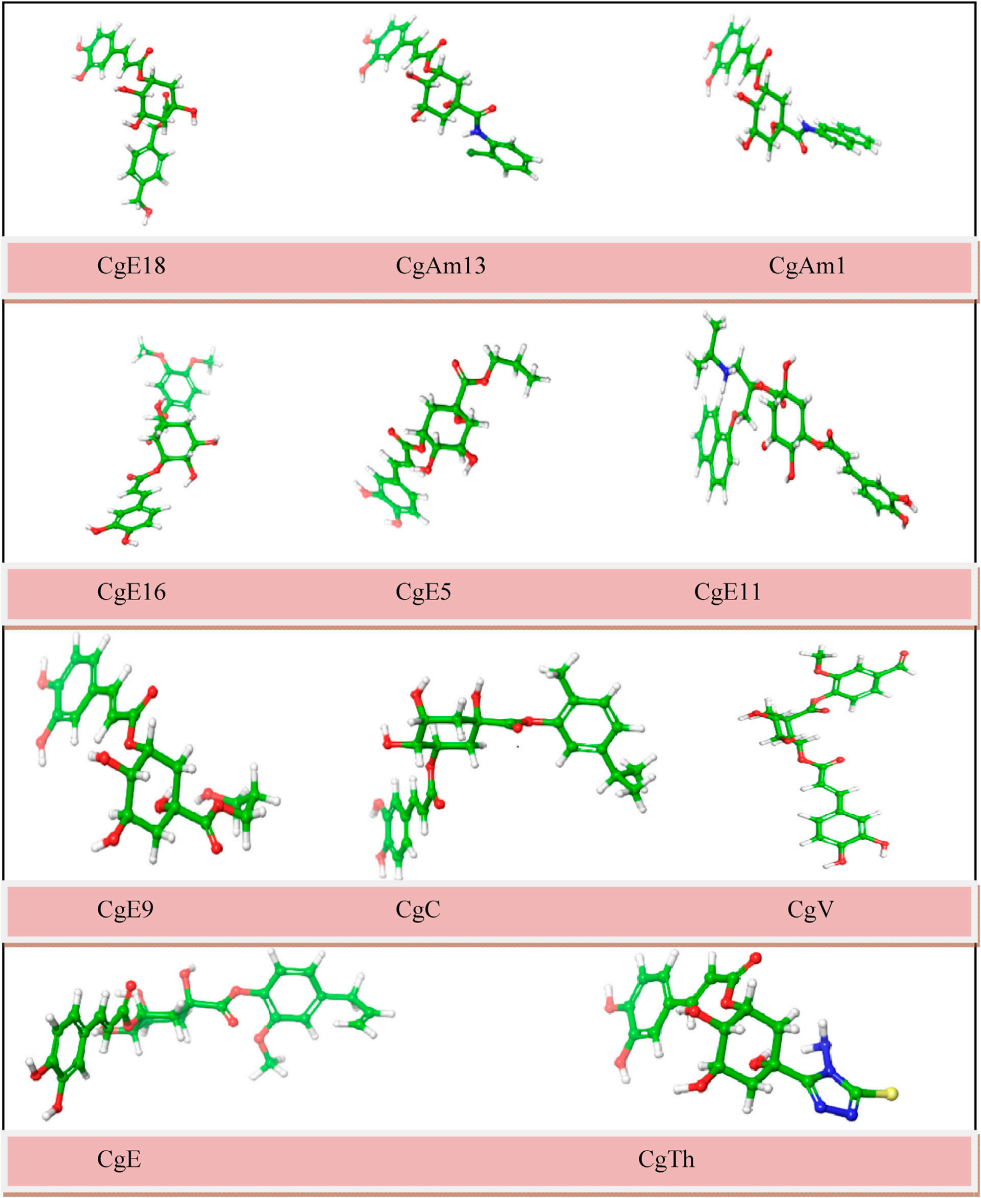
Molecular structures of optimized ligands (carbon: green; oxygen: red; hydrogen: white; nitrogen: blue; sulfur: yellow).

To obtain the most appropriate conformation and minimum attainable ground state, energy optimization of ligands is done by altering the atoms of a molecule. Optimization of ligands is required before performing molecular docking to obtain precise results. The optimized chemical figures are shown in Figure 2.

### 3.3 PASS

The online tool PASS was used to predict the antiviral, antibacterial, antifungal, and antineoplastic effectiveness of derivatives, as developed by SAR studies (Akash et al., 2022). The following acquisition PASS values were obtained: Pa score 0.383–0.479 for antiviral, 0.222–0.505 for antibacterial, 0.428–0.638 for antifungal, and 0.469–0.802 for antineoplastic. It is seen that the probability of a compound being active, or its Pa score, is greater for antineoplastic activity. Hence, breast cancer is selected as a target disease to perform other related studies (Table 2).

**TABLE 2:** Biological activity spectrum of ligands according to the PASS tool.

Compound	Antiviral		Antibacterial		Antifungal		Antineoplastic	
	Pa	Pi	Pa	Pi	Pa	Pi	Pa	Pi
CgE18	0.338	0.026	0.505	0.016	0.618	0.017	0.788	0.013
CgAm13	0.421	0.040	0.475	0.019	0.428	0.044	0.802	0.012
CgAm11	0.395	0.096	0.439	0.023	0.464	0.037	0.745	0.019
CgE16	0.465	0.007	0.482	0.018	0.589	0.020	0.798	0.012
CgE5	0.473	0.006	0.437	0.023	0.560	0.023	0.728	0.022
CgE11	0.403	0.087	0.222	0.100	0.491	0.032	0.543	0.059
CgE9	0.473	0.006	0.468	0.020	0.601	0.018	0.746	0.019
CgC	0.479	0.026	0.481	0.018	0.627	0.016	0.622	0.040
CgV	0.441	0.010	0.529	0.014	0.638	0.015	0.785	0.014
CgE	0.443	0.009	0.500	0.016	0.635	0.015	0.732	0.021
CgTh	0.383	0.112	0.331	0.049	0.579	0.021	0.469	0.081

*Pa, probability of activity; Pi, probability of inactivity.

### 3.4 Pharmacokinetic and druglikeness studies

Pharmacokinetics studies how the body interacts with administered drugs for the entire duration of exposure, and prediction of pharmacokinetics is essential to avoid the last step failure of the drug discovery process. The QikProp module of the Maestro suit was utilized to predict the ADMET profile of derivatives, and the selected properties are presented in Table 3. Lower lipophilicity of a molecule is one reason behind poor bioavailability, which in turn is a substantial reason that a drug might fail by not reaching the target site. Therefore, the estimation of ADME properties is considered important to lessen the probability of possible problems that could arise after the invention of the drug. Evaluation of different pharmacokinetic parameters was done, including lipophilicity (QPlogP o/w), predicted aqueous solubility (QPlogS), predicted IC_50_ value (QPHERG), QPPCaco cell permeability, QPlogBB, QPPMDCK, QPlogKp, QPlogKhsa, human oral absorption (HOA), %HOA, CNS active/inactive, and Lipinski’s rule of five. The table indicates that all compounds showed a high human oral absorption percentage. Results revealed that the ADME parameters of each ligand were within the bounds of the satisfactory range.

**TABLE 3:** QikProp simulation studies of selected chlorogenic acid derivatives.

Molecule	CNS	QPlogP o/w	QPlogS	CIQPlogS	QPlog HERG	QPPCaco	QPlogBB	QPPMDCK	QPlogKp	QPlogKhsa	Human oral absorption	Human oral absorption (%)	Rule of five	Rule of three
CgE18	−2	0.935	−4.557	−4.776	−6.839	5.881	−4.262	1.92	−5.457	−0.431	2	33.235	1	2
CgAm13	−2	1.92	−5.185	−5.513	−7.407	22.976	−3.305	8.377	−4.111	−0.145	2	49.595	1	1
CgAm11	−2	1.504	−4.626	−4.985	−6.629	26.851	−2.946	21.214	−4.36	−0.346	2	48.369	1	0
CgE16	−2	1.813	−5.05	−5.487	−6.567	16.915	−3.688	6.016	−4.756	−0.201	2	33.629	2	2
CgE5	−2	0.763	−3.749	−3.651	−5.665	17.465	−3.285	6.228	−5.212	−0.452	2	53.642	0	1
CgE11	−2	3.019	−5.559	−6.049	−8.685	4.358	−3.749	1.537	−5.844	0.238	1	17.187	3	2
CgE9	−2	−0.008	−3.003	−3.315	−5.532	11.086	−3.566	3.81	−5.411	−0.746	2	32.638	1	1
CgC	−2	2.367	−5.551	−5.502	−6.491	29.152	−3.236	10.835	−4.485	0.046	2	67.021	0	1
CgV	−2	0.421	−4.302	−4.571	−6.504	4.639	−4.243	1.486	−5.904	−0.55	2	28.379	1	1
CgE	−2	2.136	−5.152	−5.501	−6.703	20.949	−3.541	7.581	−4.429	−0.114	2	50.139	1	2
CgTh	−2	−1.142	−1.914	−2.539	−6.074	1.594	−2.931	1.415	−8.188	−0.838	1	0	2	1

QPlogP o/w (predicted octanol/water partition coefficient) −2.0 to 6.5. QPlogS (predicted aqueous solubility) −6.5 to 0.5. CIQPlogS (conformation-independent predicted aqueous solubility) −6.5 to 0.5. QPlog HERG (predicted IC_50_ value for blockage of HERG K+ channels) concern below −5. QPPCaco (predicted apparent Caco-2 cell permeability in nm/sec) Caco— <25 poor; >500 great. QPlogBB (predicted brain–blood barrier partition coefficient) −3.0 to 1.2. QPPMDCK (predicted apparent MDCK cell permeability in nm/sec) < 25 poor; >500 great. QPlogKp (predicted skin permeability) −8.0 to −1.0. QPlogKhsa (prediction of binding to human serum albumin) −1.5 to 1.5. Human oral absorption (predicted qualitative human oral absorption) 1, 2, or 3 for low, medium, or high. Percent human oral absorption (predicted human oral absorption) > 80% is high; <25% is poor. CNS (central nervous system) −2 is CNS inactive; +2 is CNS active (QikProp, 2023).

The druglikeness of a compound can be predicted by Lipinski’s rule, known as the rule of five. According to this, molecular weight should be < 500, octanol/water partition coefficient should be < 5, hydrogen bond donor should be less than 6, and hydrogen bond acceptor should be between 2 and 20. The compound has more druglike properties if the rule-of-five value is near zero. In the present study, all compounds possess good druglikeness properties based on this standard. As a result, none of the compounds was discarded, and each of the compounds proved to have promising druglike properties. The oral bioavailability of the compound can also be predicted from the QikProp module by calculating Jorgensen’s rule of three, the three rules are QPlogS > −5.7, QPPCaco >22 nm/s, and primary metabolites <7. The results revealed that all ligands have good oral absorption and promising ADME properties.

### 3.5 Molecular docking

Molecular docking was performed to gain insight into the binding affinities of derivatives toward the targeted breast cancer proteins using the Schrödinger Maestro suite (Sarkar et al., 2022; Katsila et al., 2016). Ligands interact with the different types of amino acid residues in several ways, like hydrogen bond formation with important amino acid residues, hydrophobic interactions, electrostatic interactions, ionic interactions, and salt bridges in the binding pockets of targeted proteins, including 7KCD, 3ERT, 6CHZ, 3HB5, and 1U72 for breast cancer (Supporting Material tabulated in Table 4). The standard drugs taken for reference were epirubicin hydrochloride, 5-fluorouracil, and methotrexate. In the breast cancer PDB, the best docking scores for 7KCD were −11.63 kcal/mol and −10.31 kcal/mol for CgE18 and CgAm13 compounds, respectively, whereas the docking score of standard epirubicin hydrochloride was −3.85 kcal/mol, and the docking score of 5-fluorouracil was −5.25 kcal/mol. The maximum docking score for 3ERT was −10.77 kcal/mol for CgE11, while epirubicin hydrochloride was −6.4 kcal/mol, and 5-fluorouracil was −3.43 kcal/mol. The maximum docking score for 6CHZ was −9.11 kcal/mol for CgE11, while epirubicin hydrochloride was −8.76 kcal/mol, and 5-fluorouracil was −3.73 kcal/mol. The maximum docking scores for 3HB5 were −14.15 kcal/mol and −12.91 kcal/mol for CgE18 and CgE11, respectively, whereas epirubicin hydrochloride was −10.5 kcal/mol, and 5-fluorouracil was −5.29 kcal/mol. The maximum docking scores for 1U72 were −12.90 kcal/mol and −11.90 for CgE18 and CgAm13, whereas the standard drug methotrexate was −13.7 kcal/mol docking score. The designed ligands demonstrate enhanced docking scores against the modeled target compared to both the standard drugs under investigation and the lead compound, chlorogenic acid. This finding suggests exciting opportunities for further exploration.

**TABLE 4:** Chlorogenic acid derivatives docked against breast cancer PDB IDs 7KCD, 3ERT, 6CHZ, 3HB5, and 1U72, indicating docking score, nature of the interaction, and amino acids involved in interaction in the active site.

Compound	PDB ID	Docking score (kcal/mol)	Nature of interaction	Amino acid residues in the active site
CgE18	7KCD	−11.63	H-Bond interaction	Glu353 and Asn532
			Pi–pi stacking	Phe404
			Hydrophobic interaction	Leu525, Trp383, Leu384, Met421, Leu387, Ile424, Met388, Phe425, Phe404, Leu391, Leu428, Met343, Leu346, Val533, Val534, Pro535, Leu539, Ala350, and Leu354
	3ERT	−9.26	H-bond interaction	Asp351 and Cys530
			Hydrophobic interaction	Leu428, Phe404, Met343, Leu391, Ile424, Leu346, Met421, Ala350, Leu525, Leu354, Leu539, Leu536, Pro535, Val534, Val533, and Cys530
	6CHZ	−7.17	H-bond interaction	Two hydrogen bonds with Glu353, Arg394, and Asp351
			Hydrophobic interaction	Phe404, Leu391, Ile424, Leu428, Met388, Met421, Leu387, Met522, Leu384, Leu525, Trp383, Met343, Leu346, Leu349, and Ala350
	3HB5	−14.15	H-bond interaction	Thr190, Gly92, Ser12, two hydrogen bonds with Gly94, and two hydrogen bonds with Gly186
			Pi–pi stacking	Phe192
			Pi–cation	Arg37
			Hydrophobic interaction	Tyr155, Val143, Cys185, Pro187, Val188, Phe226, Val188, Phe192, Leu93, Cys10, Ala91, Ile14, and Leu16
	1U72	−12.90	H-bond interaction	Phe31, Val115, Ala9, Lys55, and Glh30
			Hydrophobic interaction	Phe34, Phe31, Trp24, Val115, Leu22, Ala9, Val8, Ile7, Tyr121, Ile16, Ile60, Pro61, and Leu67
CgAm13	7KCD	−10.31	H-bond interaction	Asp351 and Ser530
			Hydrophobic interaction	Leu525, Met528, Val533, Val534, Pro535, Leu539, Leu354, Ala350, Trp383, Leu384, Leu346, Leu387, Met388, Met343, Leu391, Leu428, Phe404, Phe425, Ile424, and Met421
	3ERT	−7.8	H-bond interaction	Asp351 and Ala350
			Hydrophobic interaction	Leu391, Phe404, Met388, Leu428, Leu387, Met343, Met421, Leu384, Trp383, Leu346, Leu525, Ala350, Leu354, Val533, Val534, Leu536, and Leu539
	6CHZ	−6.5	Pi–pi stacking	Phe404
			Hydrophobic interaction	Leu539, leu536, Leu525, Met421, Leu428, Ile424, Phe404, Met343, Leu391, Leu346, Met388, Leu387, Leu349, Ala350, Leu384, Trp383, and Leu354
	3HB5	−7.16	H-bond interaction	Gly186, Val188, Ile14, Gly15, Ser12, Ser11, Asn90, and two hydrogen bonds with Gly92
			Pi–cation	Arg37
			Hydrophobic interaction	Tyr155, Cys185, Val188, Phe226, Phe192, Leu16, Ile14, Cys10, Val113, Val66, Leu64, Ala91, and Leu93
	1U72	−11.90	H-bond interaction	Glh30 and Val115
			Pi–cation	Arg70
			Hydrophobic interaction	Phe34, Phe31, Val115, Ala9, Val8, Ile7, Tyr121, Ile16, Leu22, Trp24, and Ile60
CgAm11	7KCD	−9.95	H-bond interaction	Asn532
			Pi–pi stacking	Phe404
			Hydrophobic interaction	Leu525, Val533, Leu539, Pro535, Leu354, Trp383, Leu384, Ala350, Leu349, Leu382, Met388, Leu346, Leu391, Met393, Phe404, Leu428, Phe425, Ile424, and Met421
	3ERT	−8.5	H-bond interaction	Asp351
			Hydrophobic interaction	Tyr526, Leu525, Met421, Ile424, Cys530, Val533, Val534, Pro535, Leu536, Leu354, Ala350, Trp383, Leu384, Leu346, Leu387, Met388, Met343, Leu428, Leu391, Phe404, and Ile424
	6CHZ	−6.5	Pi–pi stacking	Phe404
			Hydrophobic interaction	Leu428, Ile424, Met421, Leu525, Cys530, Ala350, Leu349, Trp383, Leu384, Leu536, Leu387, Leu536, Leu387, Leu346, Met388, Phe404, Met343, and Leu391
	3HB5	−11.45	H-bond interaction	Gly141, Ser12, Ser11, Gly92, Arg37, and Thr140
			Hydrophobic interaction	Cys185, Phe192, Ile14, Cys10, Ala91, Leu93, Val113, Val66, Leu64, and Leu36
	1U72	−11.89	H-bond interaction	Ser118, Thr146, and two hydrogen bonds with Glh30
			Pi–pi stacking	Phe34
			Halogen bond	Gly117, Lys55, and Thr56
			Hydrophobic interaction	Tyr33, Trp24, Leu22, Ile16, Val115, Phe34, Val8, Ala9, Ile7, Val120, and Tyr121
CgE16	7KCD	−7.27	H-bond interaction	Asp351 and Ser530
			Hydrophobic interaction	Leu525, Met528, Val533, Val534, Pro535, Leu391, Met343, Leu346, Met388, Leu387, Leu346, Phe425, Phe404, Ile424, Leu384, Trp383, Ala350, Leu354, and Leu539
	3ERT	−9.49	H-bond interaction	Asp351
			Hydrophobic interaction	Trp383, Leu384, Met421, Leu387, Met388, Ile424, Leu428, Leu391, Met343, Phe404, Leu346, Ala350, Leu354, Met528, Tyr526, Leu525, and Met522
	6CHZ	−1.5	H-bond interaction	Two hydrogen bonds with Glu353, Arg391, and Hie524
			Hydrophobic interaction	Leu391, Phe404, Met421, Leu428, Met388, Leu387, Ile421, Leu384, Trp383, Leu525, Met522, Met343, Leu346, Leu349, Ala350, and Leu354
	3HB5	−10.40	H-bond interaction	Val188, Ser12, Val66, Leu64, and Thr190
			Pi–cation	Arg37
			Hydrophobic interaction	Cys185, Pro187, Val188, Phe192, Ile14, Val113, Val66, Leu64, Leu36, Leu93, Ala91, Tyr155, Phe226, and Val143
	1U72	−11.83	H-bond interaction	Glh30, Ile7, Ala9, Lys55, and Thr146
			Hydrophobic interaction	Leu62, Phe34, Phe31, Ile7, Trp24, Val8, Ala9, Leu22, Tyr121, Ile16, Val115, Ile60, and Pro61
CgE5	7KCD	−8.23	H-bond interaction	Asp351
			Hydrophobic interaction	Leu354, Ala350, Pro535, Leu349, Val533, Leu525, Phe404, Leu346, Met343, Leu428, Leu391, Met388, Leu387, Ile424, Met421, Leu384, and Trp383
	3ERT	−7.9	H-bond interaction	Asp351
			Hydrophobic interaction	Met528, Leu525, Leu384, Trp383, Cys530, Val533, Leu536, Ala350, Leu349, Leu346, Phe404, Met343, Leu391, Met388, and Leu387
	6CHZ	−6.12	H-bond interaction	Asp351
			Hydrophobic interaction	Ile424, Leu346, Met421, Leu349, Ala350, Leu354, Leu536, Trp383, Leu525, Leu525, Leu384, Phe404, Leu387, Met388, Leu428, and Leu391
	3HB5	−5.35	H-bond interaction	Thr190, Asn90, Gly15, Gly141, Lys159, and Thr190
			Hydrophobic interaction	Tyr155, Cys185, Leu162, Leu16, Ile14, Ala91, Cys10, Leu36, Phe192, and Ala191
	1U72	−11.65	H-bond interaction	Asp21, Glh30, Thr146, and two hydrogen bonds with Val115
			Hydrophobic interaction	Leu22, Trp21, Val115, Phe31, Tyr121, Phe34, Phe31, Ile7, Val8, Ala9, and Ile16
CgE11	7KCD	−4.3	H-bond interaction	Asp351, Asn532, Ser530, and Ser341
			Hydrophobic interaction	Val418, Met342, Met343, Met528, Val533, Pro535, Leu354, and Leu539
	3ERT	−10.77	H-bond interaction	Asp351
			Pi–pi stacking	Tyr526
			Hydrophobic interaction	Met522, Leu525, Met528, Cys530, Val533, Val534, Pro535, Leu536, Leu539, Met421, Ile424, Phe404, Leu428, Met343, Leu391, Met388, Leu346, Ala350, Leu384, Trp383, and Leu354
	6CHZ	−9.11	H-bond interaction	Asp351
			Hydrophobic interaction	Met388, Leu387, Met343, Leu428, Phe404, Leu525, Ile424, Tyr526, Met421, Cys530, Val533, Val534, Pro535, Leu536, Leu539, Leu354, Ala354, Leu346, Leu384, and Trp383
	3HB5	−12.91	H-bond interaction	Gly186, Gly15, Gly92, Ser12, and Thr190
			Pi–cation	Two bonds with Lys195
			Hydrophobic interaction	Tyr155, Val143, Cys185, Pro187, Val188, Phe226, Ala191, Phe192, Val196, Leu93, Ala91, Cys10, Ile14, and Leu16
	1U72	−11.44	H-bond interaction	Glh30 and two hydrogen bonds with Asp21
			Pi–pi stacking	Phe31 and Phe34
			Salt bridge	Asp21
			Hydrophobic interaction	Ile7, Val8, Ala9, Val115, Tyr121, Ile16, Leu22, Trp24, Pro24, Leu67, Phe34, Phe31, Pro61, and Ile60
CgE9	7KCD	−8.7	H-bond interaction	Glu353, Arg394, and two hydrogen bonds with Asn532
			Hydrophobic interaction	Val533, Val534, Pro535, Leu539, Leu354, Ala350, Leu349, Leu391, Phe404, Met388, Leu428, Leu387, Leu346, Met343, Leu525, Trp383, Leu384, Phe425, Ile424, and Met421
	3ERT	−8.82	H-bond interaction	Cys530 and two hydrogen bonds with Asp351
			Hydrophobic interaction	Leu354, Ala350, Leu346, Met421, Met343, Phe404, Leu391, Leu428, Met388, Leu387, Leu525, Tyr526, Leu384, Trp383, Met528, Cys530, Val533, and Leu536
	6CHZ	−7.04	H-bond interaction	Glu353 and Arg394
			Hydrophobic interaction	Trp383, Leu384, Leu387, Met388, Leu391, Ala350, Leu349, Phe404, Leu346, Met343, and Leu525
	3HB5	−12.11	H-bond interaction	Thr190, Ser12, Ser11, Cys10, Gly92, Arg37, and two hydrogen bonds with Gly186
			Hydrophobic interaction	Phe226, Val188, Pro187, Cys185, Val143, Tyr155, Leu16, Ile14, Cys10, Ala91, and Leu93
	1U72	−11.355	H-bond interaction	Arg70, Pro66, Gln35, Asn64, Ala9, and Val115
			Hydrophobic interaction	Leu67, Pro66, Phe34, Phe31, Pro61, Ile60, Phe31, Tyr121, Val115, Ile116, Val8, Ala9, and Leu22
CgC	7KCD	−9.24	H-bond interaction	Asp351
			Hydrophobic interaction	Pro535, Val534, Val533, Met528, Leu525, Ile424, Leu391, Leu354, Leu428, Met388, Leu387, Ala350, Leu349, Leu384, Phe404, Trp383, Leu539, Leu346, and Met343
	3ERT	−9.05	H-bond interaction	Asp351
			Hydrophobic interaction	Leu354, Ala350, Trp383, Leu384, Leu346, Leu387, Met388, Met343, Leu391, Phe404, Leu539, Leu536, Pro535, Val534, Val533, Cys530, Met528, Tyr526, and Leu525
	6CHZ	−9.35	H-bond interaction	Asp351
			Hydrophobic interaction	Met342, Leu346, Leu349, Ala350, Leu354, Met522, Leu536, Leu525, Cys530,Tyr526, Trp383, Leu384, Leu387, Phe401, Met388, and Leu39
	3HB5	−9.03	H-bond interaction	Thr190, Val66, Leu64, Gly9, Asn90, Ser12, and Gly15
			Hydrophobic interaction	Tyr155, Cys185, Val188, Pro187, Phe192, Val113, Val66, Leu64, Leu93, Ala91, Cys10, Ile14, Leu16, and Phe226
	1U72	−11.355	H-bond interaction	Val115, Lys55, and Asp21
			Pi–pi stacking	Phe31 and Phe34
			Hydrophobic interaction	Phe34, Phe31, Ile60, Pro61, Leu67, Ile7, Val8, Ala9, Val115, Ile16, Tyr121, Leu22, and Trp24
CgV	7KCD	−8.15	H-bond interaction	Asp351 and Ser530
			Hydrophobic interaction	Met528, Leu525, Met343, Val533, Val534, Pro535, Leu539, Trp383, Leu384, Ala350, Ile424, Leu387, Met388, Leu428, Phe404, and Leu391
	3ERT	−8.93	H-bond interaction	Cys530
			Hydrophobic interaction	Leu525, Met528, Cys530, Val533, Leu536, Leu354, Ala350, Trp383, Leu384, Met421, Phe404, Leu428, Leu387, Ile424, Leu346, Met388, Met343, and Leu391
	6CHZ	−3.98	H-bond interaction	Two hydrogen bonds with Asp351
			Hydrophobic interaction	Leu536, Val533, Lys530, Met528, Tyr526, Leu525, Met522, Leu354, Ala350, Ile425, Leu428, Leu346, Met343, Phe404, Leu391, Met388, Leu387, Leu384, and Trp393
	3HB5	−10.40	H-bond interaction	Gly94, Asn90, Tyr155, Gly15, Ser12, and Gly9
			Hydrophobic interaction	Cys185, Pro187, Val188, Phe192, Ala91, Val196, Leu93, Tyr155, Phe226, Leu16, Ile14, and Cys10
	1U72	−10.67	H-bond interaction	Glh30 and Lys55
			Pi–pi stacking	Phe31
			Hydrophobic interaction	Leu67, Phe34, Phe31, Ile7, Val8, Ala9, Trp24, Leu22, Ile16, Tyr121, Ile60, Pro61, and Val115
CgE	7KCD	−3.4	H-bond interaction	Asp351 and Ser530
			Hydrophobic interaction	Leu525, Met528, Val533, Val534, Pro535, Leu354, Trp383, Leu384, Met421, Leu387, Ala350, Met388, Ile424, Phe404, Phe425, Leu391, Leu346, Leu428, and Met343
	3ERT	−8.93	H-bond interaction	Asp351
			Hydrophobic interaction	Leu354, Ala350, Trp383, Leu384, Leu386, Leu387, Met388, Leu391, Ile424, Leu428, Phe404, Met421, Leu536, Pro535, Val533, Cys530, Met528, Tyr526, and Leu525
	6CHZ	−3.98	H-bond interaction	Two hydrogen bonds with Glu353
			Pi–pi stacking	Phe404
			Hydrophobic interaction	Leu428, Met421, Ile424, Leu384, Trp383, Leu387, Met388, Leu391, Leu354, Leu525, Met528, Cys530, Met343, Leu346, Leu536, Leu349, Ala350, and Leu354
	3HB5	−10.40	H-bond interaction	Gly15, Gly9, Glu194, and Lys195
			Hydrophobic interaction	Phe226, Val143, Ala91, Tyr155, Cys185, Leu16, Ile14, Pro187, Val188, Ala191, Cys10, and Phe192
	1U72	−7.79	H-bond interaction	Glh30 and Phe31
			Hydrophobic interaction	Leu67, Phe34, Phe31, Ile7, Val8, Ala9, Trp24, Leu22, Ile16, Val115, Tyr121, Ile60, and Pro61
CgTh	7KCD	−8.83	H-bond interaction	Glu353 and Asn532
			Pi–pi stacking	Phe404
			Hydrophobic interaction	Met343, Leu346, Leu349, Val533, Val534, Ala350, Pro535, Leu354, Leu391, Leu428, Phe404, Met388, Leu387, Leu384, Trp383, Ile424, and Met421
	3ERT	−8.25	H-bond interaction	Asp351 and Glu353
			Hydrophobic interaction	Ile424, Met421, Leu525, Leu536, Leu354, Ala350, Leu349, Trp383, Leu384, Leu387, Met388, Phe404, Leu391, Leu346, and Met343
	6CHZ	−2.17	H-bond interaction	Two hydrogen bonds with Glu353, Arg394, and Hie524
			Pi–pi stacking	Phe404
			Hydrophobic interaction	Leu391, Phe404, Met388, Leu387, Met421, Leu428, Ile424, Leu384, Trp383, Leu525, Met343, Leu346, Leu349, and Ala350
	3HB5	−9.4	H-bond interaction	Gly141, Val66, Leu64, Ser12, and Thr190
			Pi–cation	Arg37
			Hydrophobic interaction	Tyr155, Cys185, Ala91, Leu36, Leu93, Val113, Val66, Leu64, Ile14, and Phe192
	1U72	−7.79	H-bond interaction	Asn64 and Ala9
			Salt bridge	Lys55
			Hydrophobic interaction	Leu67, Pro61, Ile60, Val115, Ile16, Phe31, Phe34, Ile7, Val8, Ala9, Tyr121, Trp24, Pro23, Leu22, and Ile16
Chlorogenic acid	7KCD	−5.26	H-bond interaction	Asn532
			Hydrophobic interaction	Leu525, Leu346, Met421, Met342, Ile424, Phe425, Leu428, Leu391, Phe404, Met388, Leu387, Leu384, Trp383, Pro535, Leu354, Val534, and Val533
	3ERT	−7.9	H-bond interaction	Two H-bonds with Asp351
			Hydrophobic interaction	Trp383, Leu525, Leu384, Leu387, Leu391, Phe404, Met343, Ile424, Leu346, Met421, Ala350, Leu354, Leu536
	6CHZ	−6.11	H-bond interaction	Cys530 and two H-bonds with Asp351
			Hydrophobic interaction	Leu525, Met522, Met388, Leu387, Leu384, Trp383, Cys530, Leu346, Phe404, Leu536, Leu349, and Ala350
	3HB5	−10.22	H-bond interaction	Hie221, Ser142, and two H-bonds with Glu282
			Hydrophobic interaction	Leu262, Phe259, Val225, Phe259, Val225, Phe226, Val143, Cys185, Pro187, Val188, Tyr155, Met193, Phe192, Leu149, Met279, Tyr218, and Val283
	1U72	−10.2	H-bond interaction	Lys55, Ala9, two H-bonds with Val115, and two H-bonds with Thr146
			Hydrophobic interaction	Ile16, Tyr121, Val115, Ile7, Val8, Ala9, Phe31, Phe34, Trp24, and Leu22
Methotrexate	1U72	−13.7	H-bond interaction	Two H-bonsd with Glh30, Val115, Ile 7, and two H-bonds with Asn64
			Salt bridge	Gln35 and Arg70
			Hydrophobic interaction	Tyr121, Val115, Ile7, Val8, Ala9, Phe31, Phe34, Tyr33, Ile60, Pro61, and Leu67
Epirubicin hydrochloride	7KCD	−3.85	H-bond interaction	Asn532
			Hydrophobic interaction	Val533, Val534, Pro535, Trp383, Leu539, Met543, Val355, and Leu354
	3ERT	−6.4	H-bond interaction	Asp351 and Leu525
			Hydrophobic interaction	Leu354, Ala350, Trp383, Leu536, Val533, Cys530, Met528, Tyr526, Leu525, and Met522
	6CHZ	−8.76	H-bond interaction	Leu536, Glu 380, Tyr526, and Cys530
			Hydrophobic interaction	Leu536, Pro535, Val533, Cys530, Met528, Tyr526, Leu525, Met522, Ala350, Leu354, and Trp383
	3HB5	−10.5	H-bond interaction	Thr140, Lys159, Gly92, Asn90, and two hydrogen bonds with Thr190
			Hydrophobic interaction	Phe226, Tyr155, Val143, Leu162, Ala91, Phe226, Cys185, Pro187, Val188, Ala91, Ile14, Phe192, and Met93
	1U72	---	----	----
5-Fluorouracil	7KCD	−5.25	H-bond interaction	Glu353 and Arg394
			Pi–pi stacking	Phe404
			Hydrophobic interaction	Leu346, Leu349, Ala350, Phe404, Leu391, Leu428, Met388, and Leu387
	3ERT	−3.43	H-bond interaction	Glu353 and Arg394
			Hydrophobic interaction	Leu384, Leu387, Met388, Leu391, Ala350, Leu349, and Leu346
	6CHZ	−3.73	H-bond interaction	Glu353
			Hydrophobic interaction	Leu387, Met388, Leu384, Leu346, Leu349, Ala350, Phe404, and Leu391
	3HB5	−5.29	H-bond interaction	Gly9, Asn90, Gly15, and two hydrogen bonds with Gly92
			Hydrophobic interaction	

### 3.6 Binding/docking pose of ligands in the active site of the target protein

**FIGURE 3:**
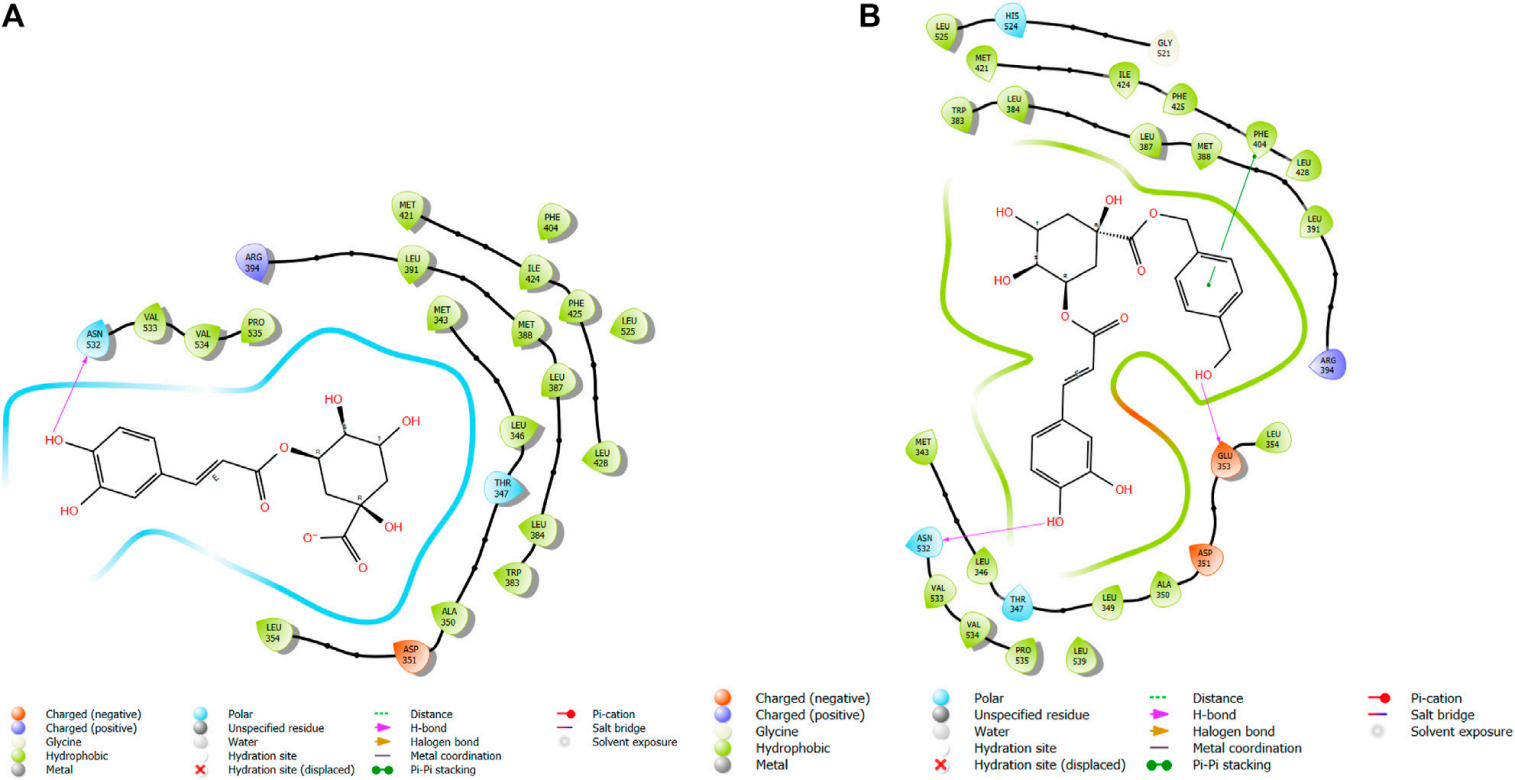
Comparative 2D view interactions of CLG [**(A)**; docking score −5.26 kcal/mol] and compound CgE18 [**(B)**; docking score −11.63 kcal/mol] with breast cancer protein (PDB ID 7KCD). CgE18 demonstrates enhanced hydrophobic character and increased binding affinity.

**FIGURE 4:**
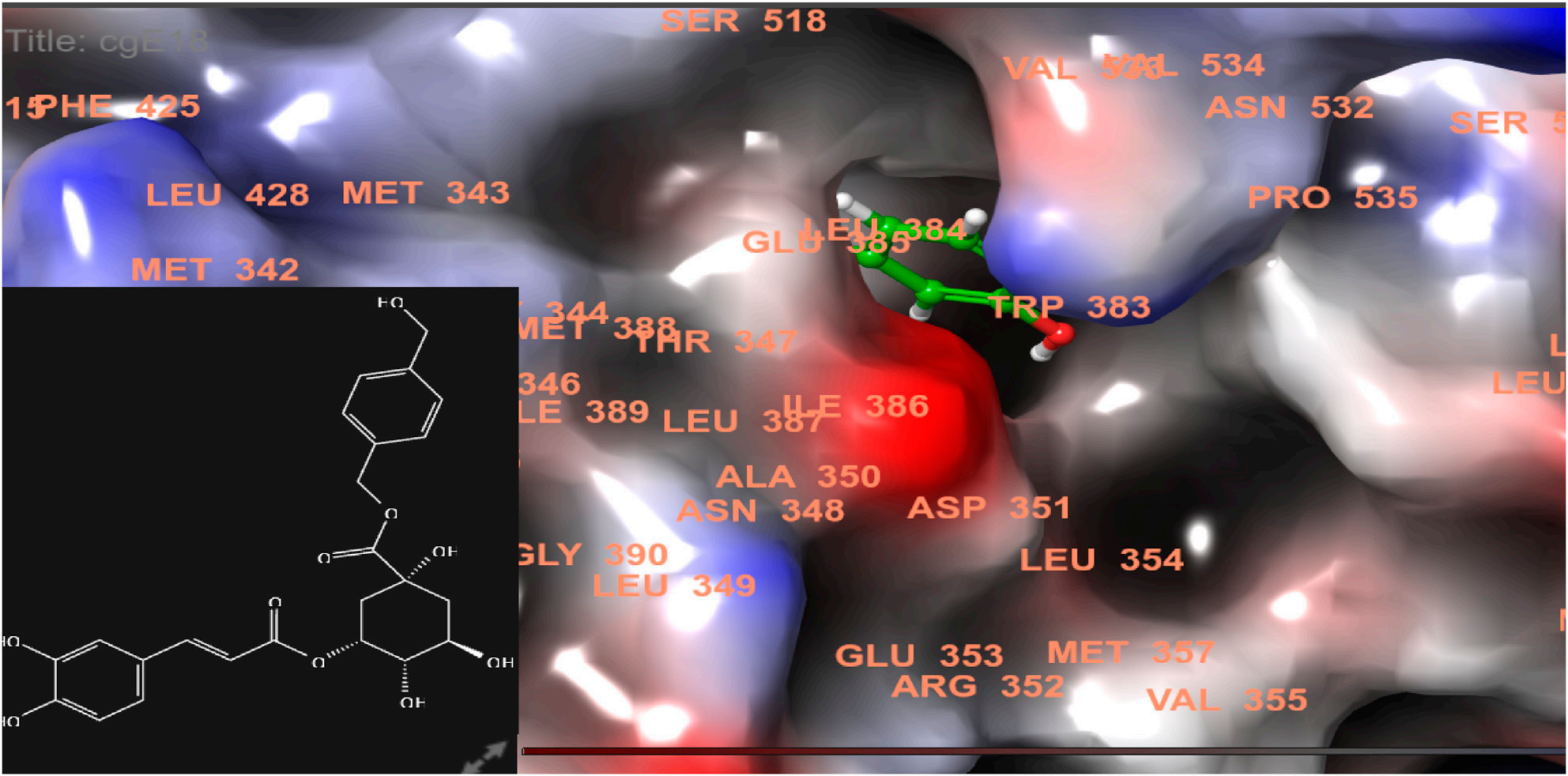
3D model of ligand CgE18 in the active pocket of the breast cancer protein receptor (PDB ID 7KCD) with amino acid residues present in proximity to the active site.

**FIGURE 5:**
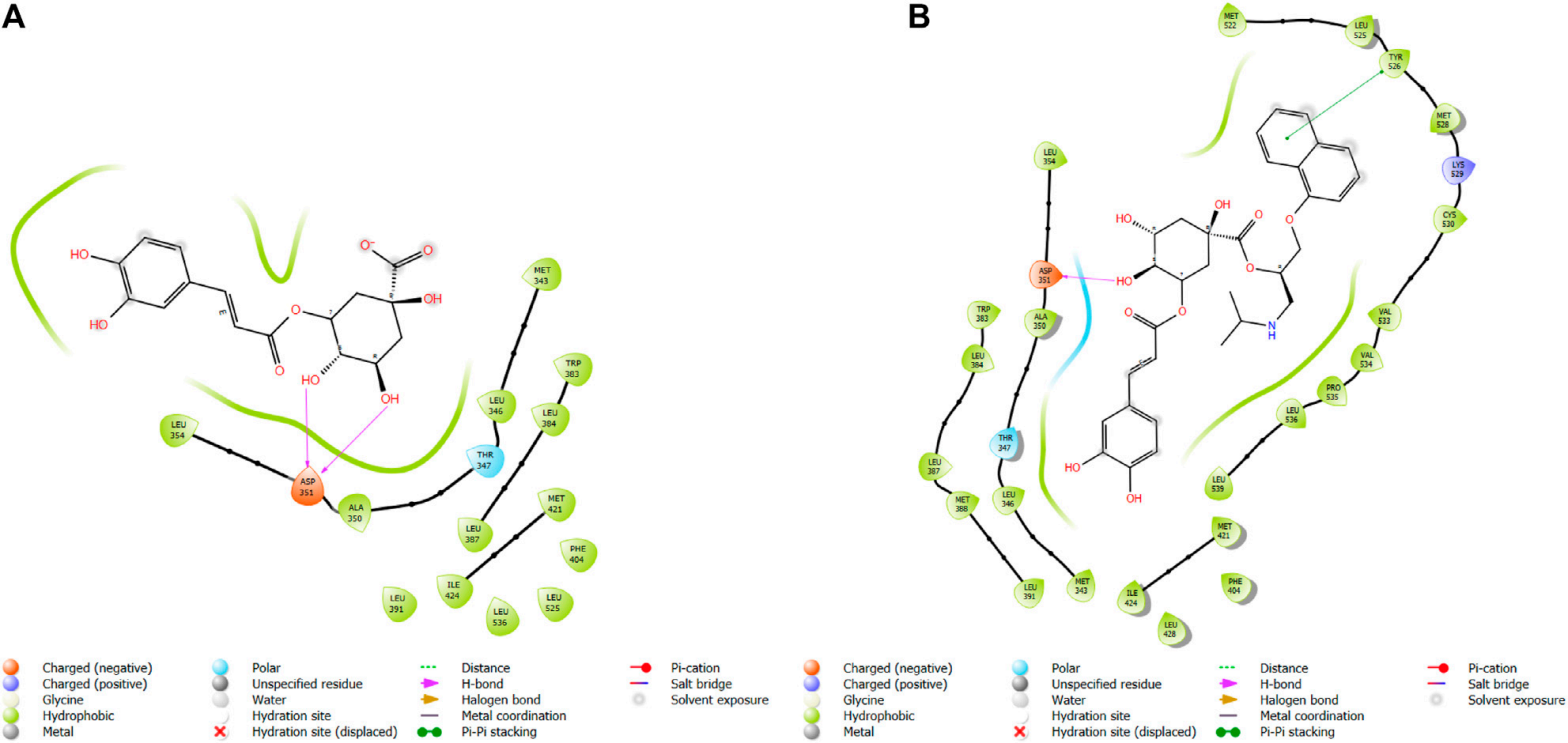
Comparative 2D view interactions of CLG [**(A)**; docking score −7.9 kcal/mol] and compound CgE11 [**(B)**; docking score −10.77 kcal/mol] with breast cancer protein (PDB ID 3ERT). CgE11 demonstrates enhanced hydrophobic character and increased binding affinity.

**FIGURE 6:**
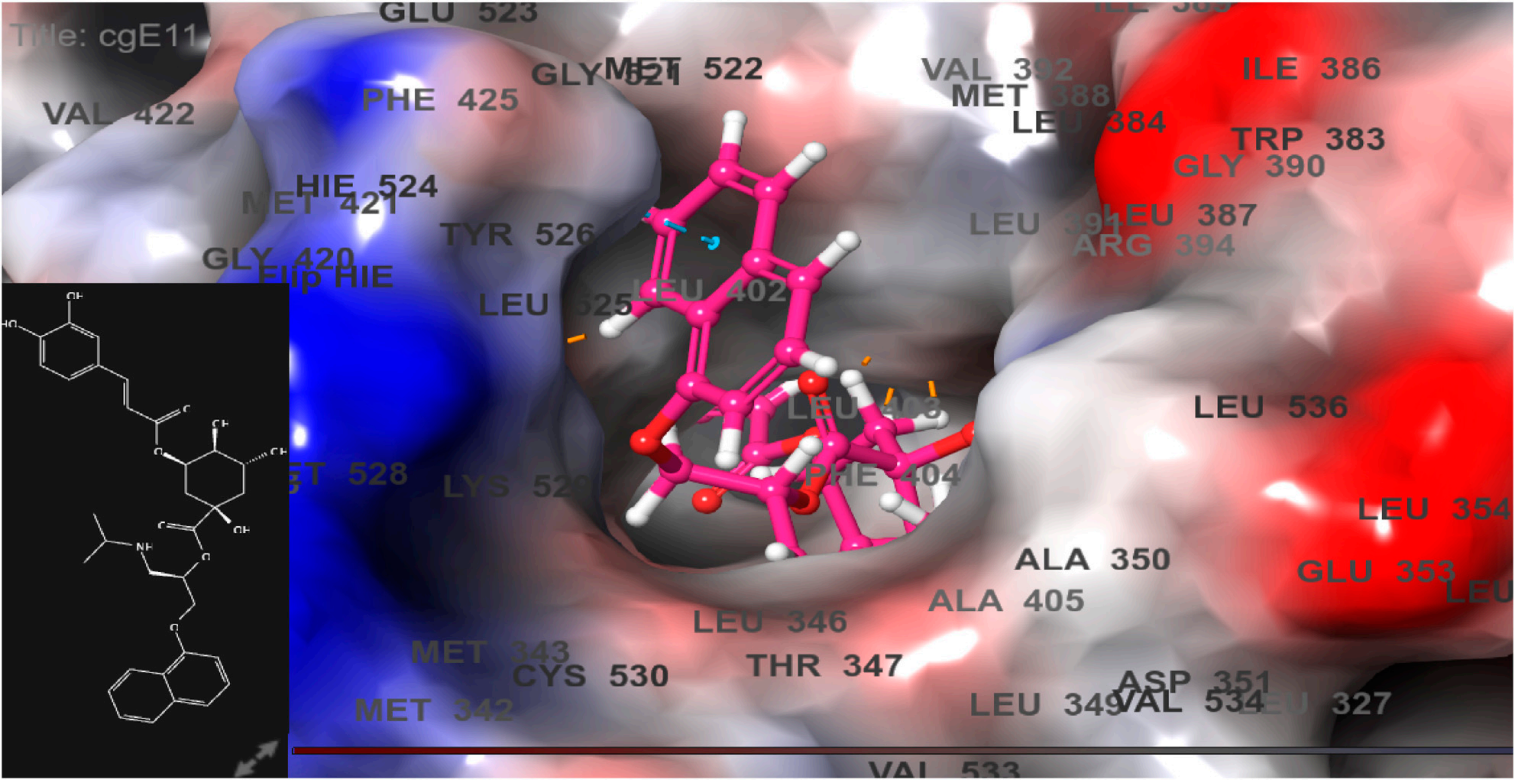
3D model of ligand CgE11 in the active pocket of the breast cancer protein receptor (PDB ID 3ERT) with amino acid residues present in proximity to the active site.

**FIGURE 7:**
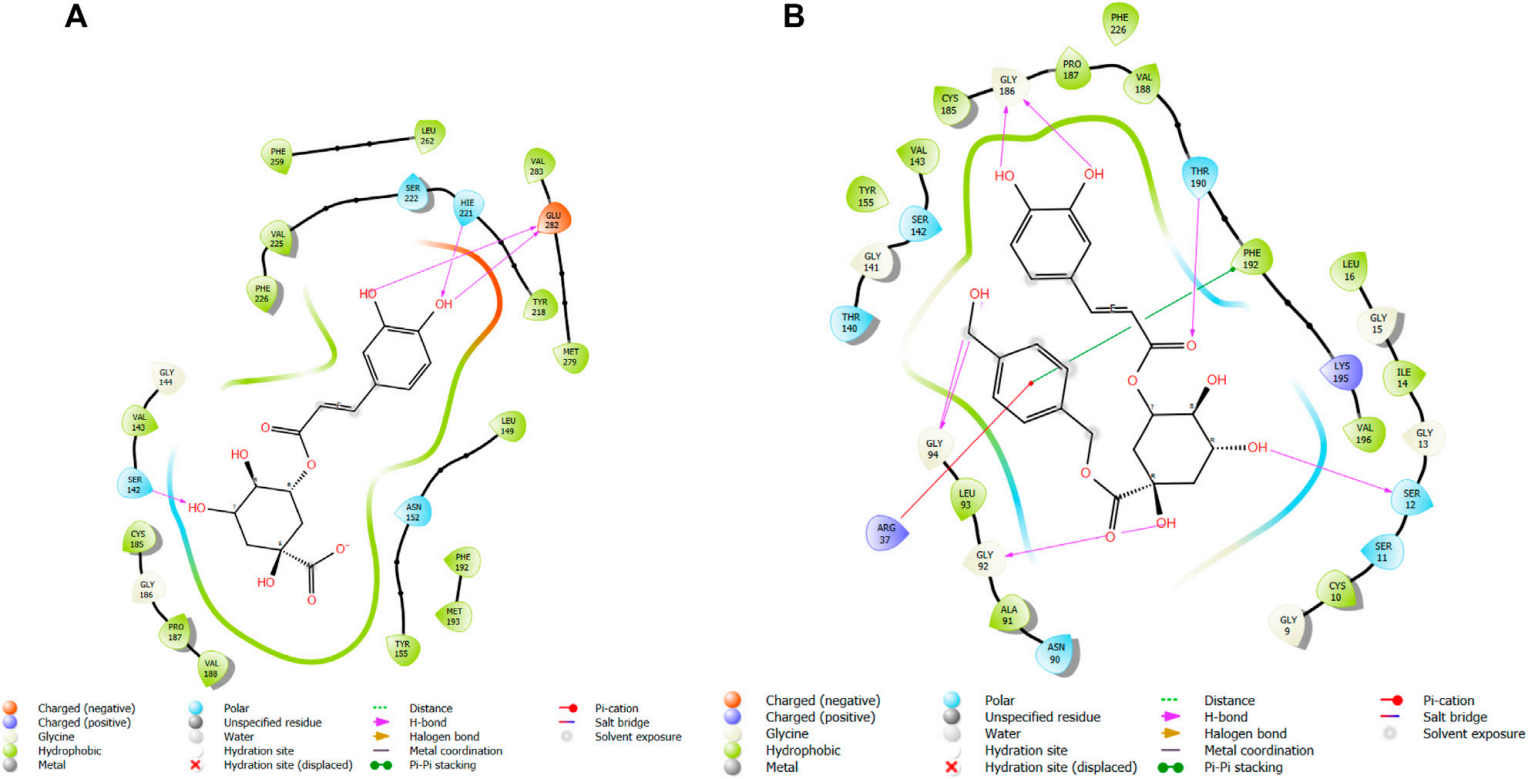
Comparative 2D view interactions of CLG [**(A)**; docking score −10.22 kcal/mol] and compound CgE18 [**(B)**; docking score −14.15 kcal/mol] with breast cancer protein (PDB ID 3HB5). CgE18 demonstrates enhanced hydrophobic character and increased binding interaction.

**FIGURE 8:**
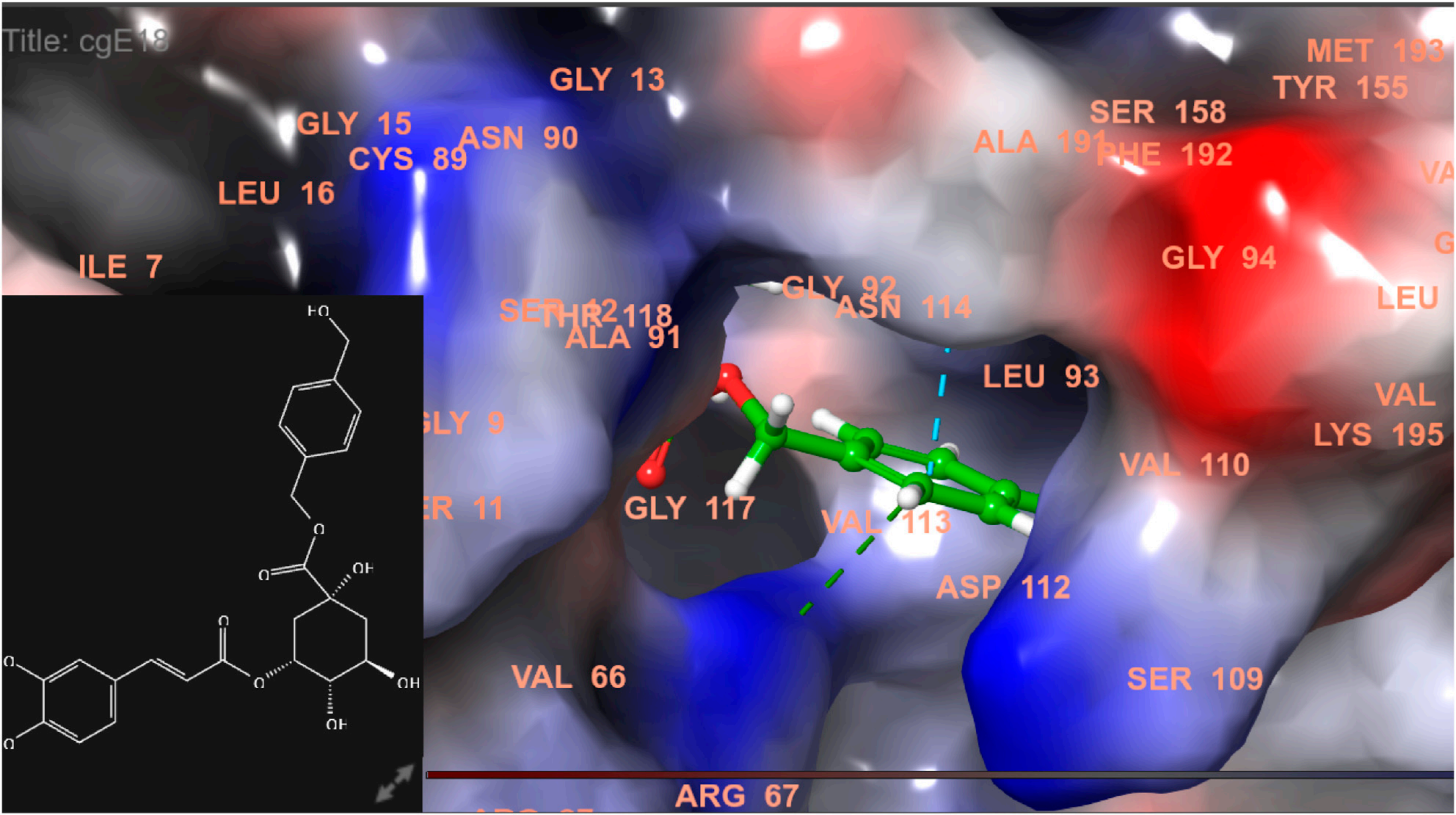
3D model of ligand CgE18 in the active pocket of breast cancer protein receptor (PDB ID 3HB5) with amino acid residues present in proximity to the active site.

**FIGURE 9:**
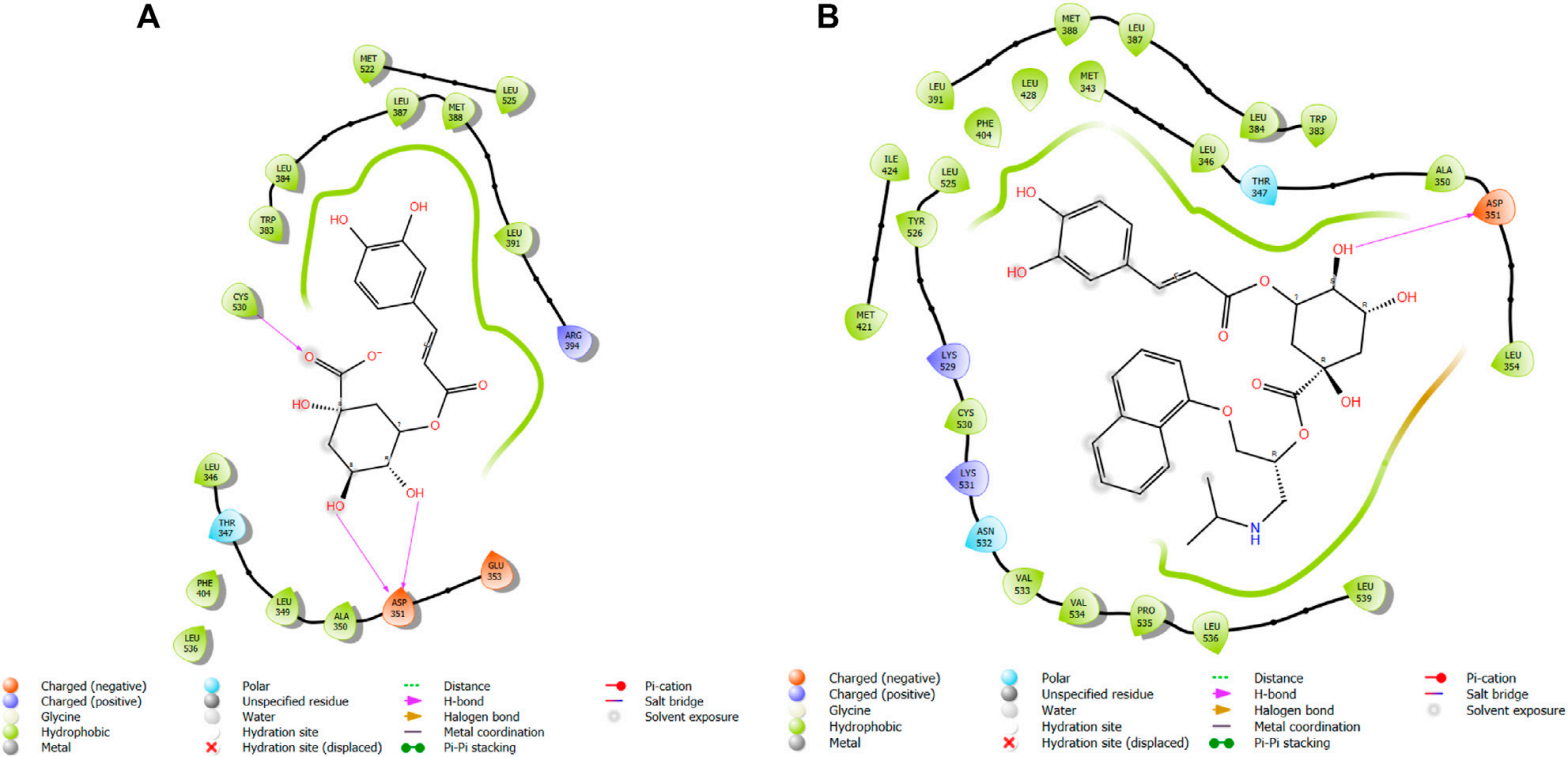
Comparative 2D view interactions of CLG [**(A)**; docking score −6.11 kcal/mol] and compound CgE11 [**(B)**; docking score −9.11 kcal/mol] with breast cancer protein (PDB ID 6CHZ). CgE11 demonstrates enhanced hydrophobic character and increased binding affinity.

**FIGURE 10:**
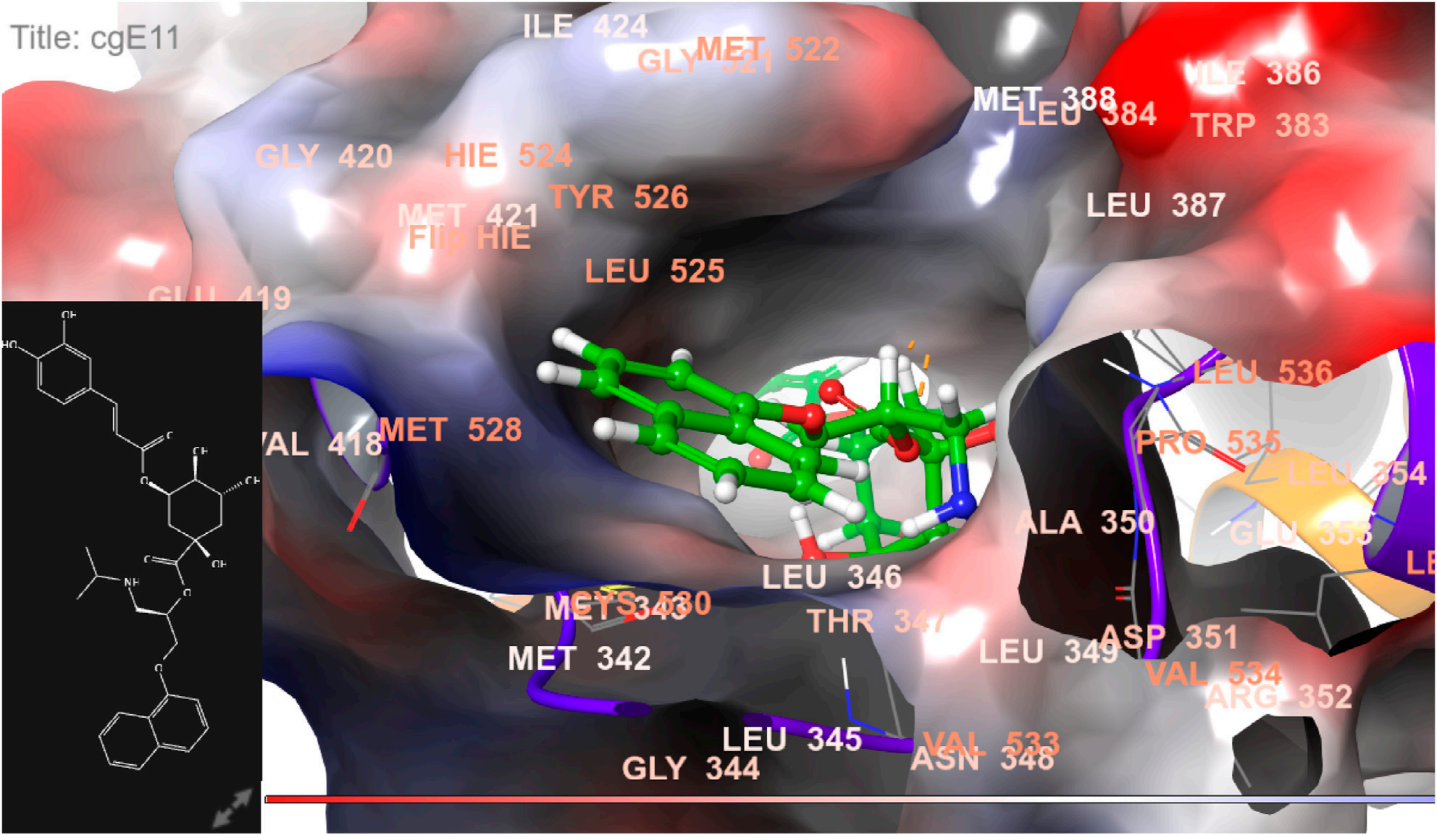
3D model of the ligand CgE11 in the active pocket of the breast cancer protein receptor (PDB ID 6CHZ) with amino acid residues present in proximity to the active site.

**FIGURE 11:**
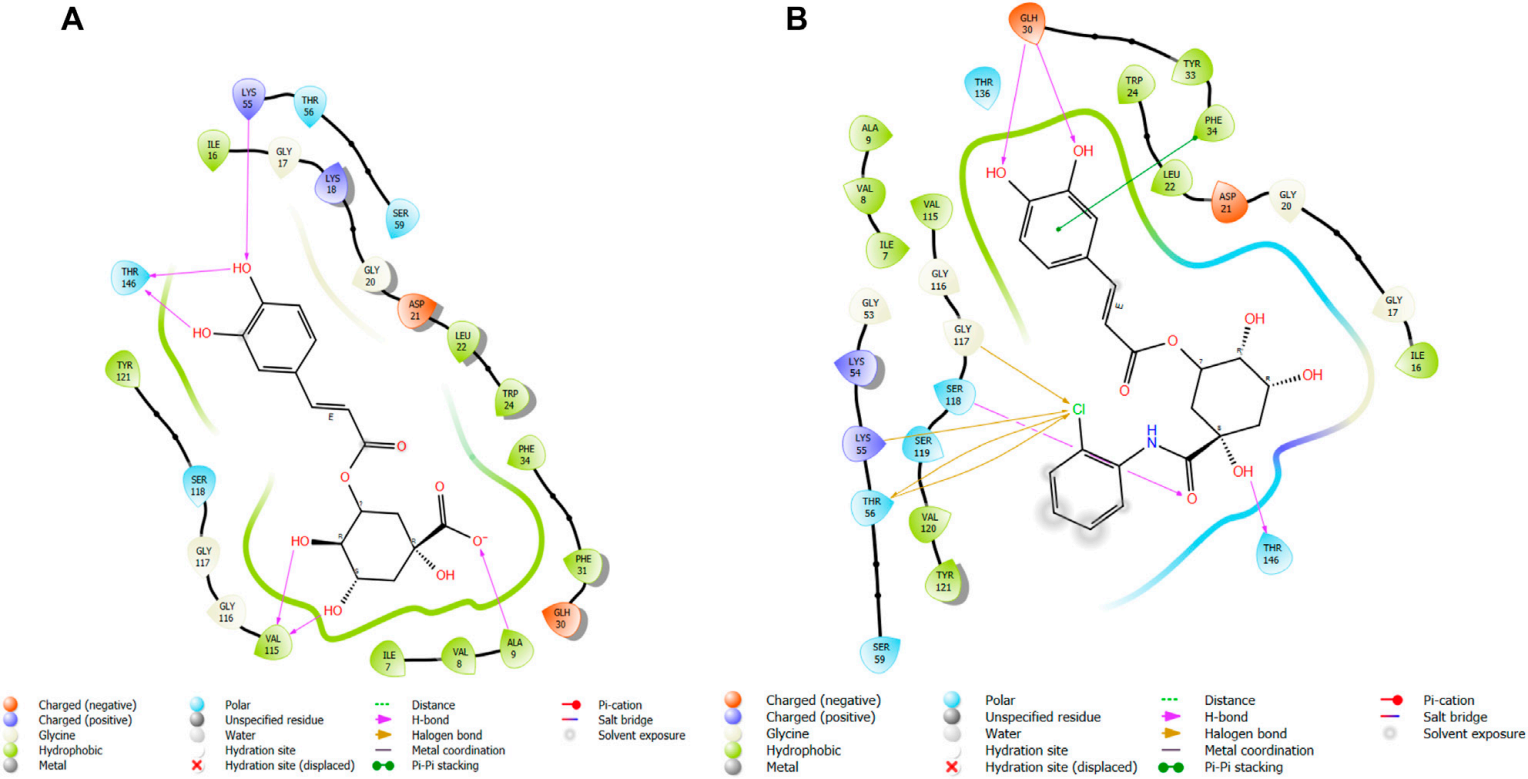
Comparative 2D view interactions of CLG [**(A)**; docking score −10.2 kcal/mol) and compound CgAm11 [**(B)**; docking score −11.89 kcal/mol] with breast cancer protein (PDB ID 1U72). CgAm11 demonstrates enhanced hydrophobic character and increased binding interaction.

**FIGURE 12:**
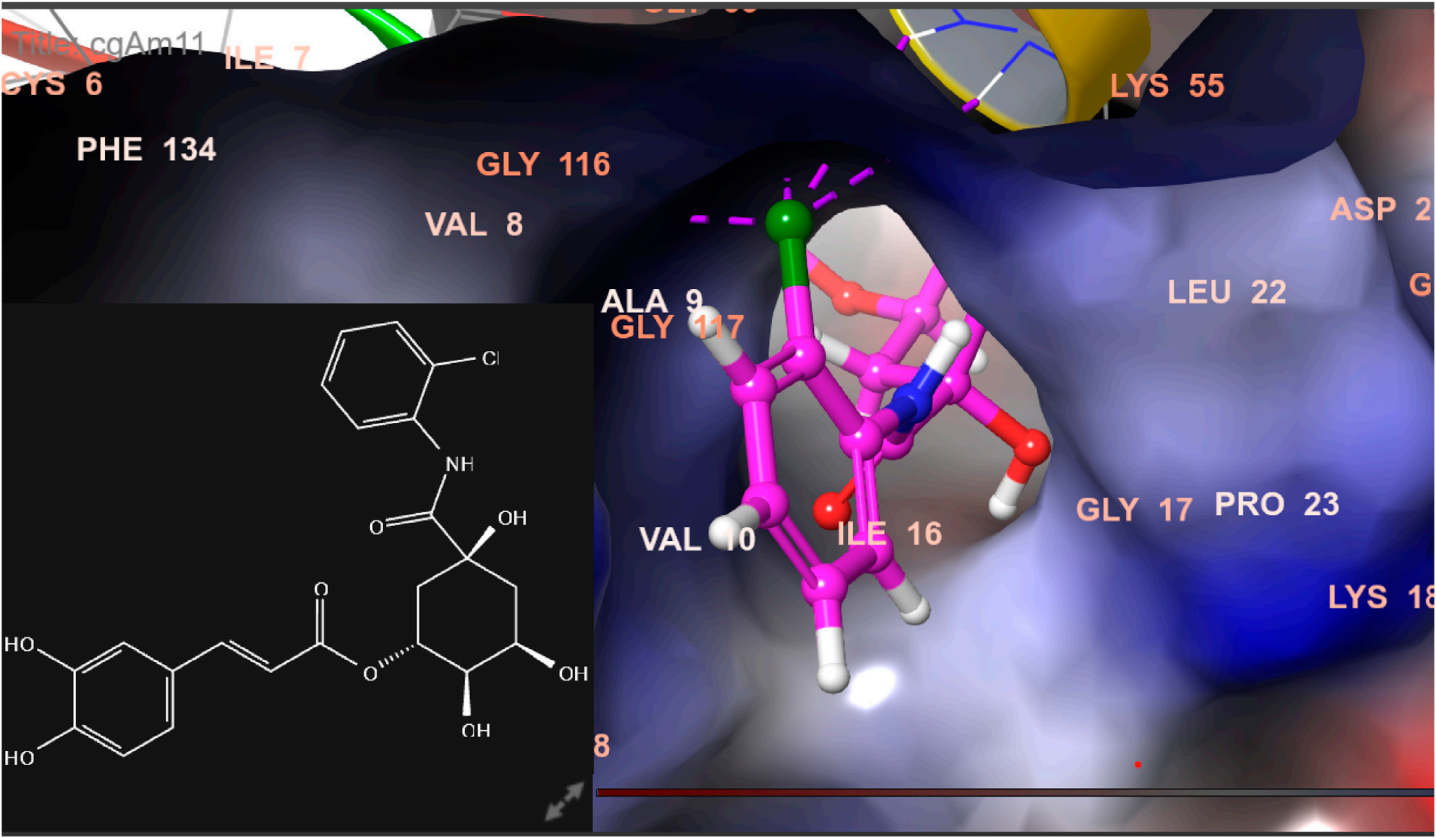
3D model of the ligand CgAm11 in the active pocket of the breast cancer protein receptor (PDB ID 1U72) with amino acid residues present in proximity to the active site.

In this study, chlorogenic acid derivatives were docked against breast cancer proteins to gain insights into the binding affinities of designed ligands with the target protein. Exhaustive analysis of ligand–protein interaction poses showed that newly designed biomolecules bind within the binding pocket of targeted proteins firmly by hydrogen bond formation, salt bridge formation, pi–pi stacking, p-cation, and hydrophobic interaction, as shown in Figures 3–12. The docking pose of the most active ligand (CgE18) with protein PDB ID 7KCD showed astonishing interactions; it interacts by two hydrogen bonds between the hydroxyl group of the ligand with residues Glu353, Asn532, pi–pi stacking with Phe404, and hydrophobic interaction with Leu525, Trp383, Leu384, Met421, Leu387, Ile424, Met388, Phe425, Phe404, Leu391, Leu428, Met343, Leu346, Val533, Val534, Pro535, Leu539, Ala350, and Leu354. Asp351, Ser530, and Phe404 are the common amino acid residues of PDB ID 7KCD that form maximum hydrogen bonds with ligands. The findings of Akash et al. (2022) support interactions of this kind and the participation of specific amino acids in binding.

The widely recognized anticancer medicines, namely, epirubicin hydrochloride and 5-fluorouracil, also exhibit binding to identical amino acid residues such as Glu353, Asn532, Phe404, and Asp351. These findings further strengthen the potential of this compound as an anticancer agent. The docking pose of the most active ligand (CgE11) with protein PDB ID 3ERT showed excellent interactions as it interacts by hydrogen bonds between the hydroxyl group of the ligand with residue Asp351, pi–pi stacking with Tyr526, and hydrophobic interaction with Met522, Leu525, Met528, Cys530, Val533, Val534, Pro535, Leu536, Leu539, Met421, Ile424, Phe404, Leu428, Met343, Leu391, Met388, Leu346, Ala350, Leu384, Trp383, and Leu354. Asp351 and Glu353 amino acid residues of PDB ID 3ERT form maximum hydrogen bonds with ligands and with the standards epirubicin hydrochloride and 5-fluorouracil and also interact with the same Asp351 and Glu353, respectively, which also validates the active site. Co-crystalized ligand tamoxifen also binds to the same site, and this finding is supported by several studies (Puranik et al., 2019; Tilak Vijay et al., 2019; Shah et al., 2022; Shtaiwi et al., 2019).

The docking pose of the most active ligand (CgE11) with protein PDB ID 6CHZ showed good interactions as it forms a hydrogen bond with Asp351 and has a hydrophobic interaction with Met388, Leu387, Met343, Leu428, Phe404, Leu525, Ile424, Tyr526, Met421, Cys530, Val533, Val534, Pro535, Leu536, Leu539, Leu354, Ala354, Leu346, Leu384, and Trp383 amino acid residues. Asp351, Glu353, and Phe404 of PDB ID 6CHZ form maximum hydrogen bonds with designed ligands; standard 5-fluorouracil also interacts with the same Glu353, which also validates the active site. The docking configuration of the most potent ligand, CgE18, with protein PDB ID 3HB5, exhibited remarkable interactions. It formed a total of seven hydrogen bonds, one with each of Thr190, Gly92, and Ser12, two hydrogen bonds with Gly94, and two with Gly186. Furthermore, it engaged in pi–pi stacking interactions with Phe192, pi–cation interactions with Arg37, and hydrophobic interactions involving Tyr155, Val143, Cys185, Pro187, Val188, Fhe226, Val188, Phe192, Leu93, Cys10, Ala91, Ile14, and Leu16 amino acid residues. Notably, the formation of pi–cation bonds with Arg37 and hydrogen bonds with Ser12, Val188, and Gly92 were the most commonly observed interactions among the designed ligands in the protein’s active site. It is worth mentioning that the standard anticancer drug Epirubicin hydrochloride also established identical hydrogen bonds with amino acid residues and shared a similar binding core, confirming the correct active site. These findings align with the research conducted by Akash et al. (2022). However, the designed compound exhibited a higher binding score than the standard, possibly due to its additional pi–pi stacking interaction with the crucial amino acid residue Phe192 and the pi–cation bond with Arg37, in addition to a greater number of hydrogen bonds.

The docking pose of the most active ligands (CgE18 and CgAm13) with protein PDB ID 1U72 showed good interactions. The CgE18 compound forms five hydrogen bonds with Phe31, Val115, Ala9, Lys55, and Glh30 and hydrophobic interactions with Phe34, Phe31, Trp24, Val115, Leu22, Ala9, Val8, Ile7, Tyr121, Ile16, Ile60, Pro61, and Leu67 amino acid residues. Hydrogen bonds with Glh30, Val115, and pi–pi stacking with Phe31 and Phe34 amino acid residues of 1U72 are common for most of the compounds. Standard methotrexate (MTX) also binds with the same amino acid residues, which validates the active site, and this finding aligns with Wang et al.’s (2017) results.

### 3.7 MM-GBSA binding free energy

The molecular mechanics-generalized born surface area (MM-GBSA) technique is a computational method that assesses the binding free energy between a ligand and receptor within a biological system. Its primary use lies in predicting the strength of binding interactions and differentiating between drugs and binders exclusively. One can expect the order of ligands, ranked by their calculated binding energies (MM-GBSA ΔG Bind), to closely align with the ranking based on experimental binding affinity. It is worth noting that a more negative value indicates stronger binding as MM-GBSA binding energies effectively represent free energies of binding. MM-GBSA binding energy calculations lie between the precise alchemical perturbation methods and empirical scoring methods regarding accuracy. These calculations involve molecular dynamics simulations of the receptor–ligand complex, providing a more dynamic and realistic view of binding interactions than purely empirical approaches [Genheden and Ryde, 2015; Sehrawat et al., 2023; Schrödinger, accessed on 4 May 2023]. Data shown in Table 5 indicate that the compounds with the highest docking scores demonstrate enhanced binding energy compared to the standard drugs MTX, epirubicin hydrochloride, and 5-fluorouracil, except for the MTX binding energy against 7KCD and 1U72. This implies that the created ligands host a heightened level of stability during interaction with target protein binding pockets.

**TABLE 5:** Comparison of binding energies between the top docked compounds and reference drugs.

PDB ID	Top docked compound [binding energy (kcal/mol)]	Epirubicin hydrochloride [binding energy (kcal/mol)]	5-Fluorouracil [binding energy (kcal/mol)]	Methotrexate [binding energy (kcal/mol)]
7KCD	−43.61(CgE18)	−25.23	−23.97	−45.03
3ERT	−50.99 (CgE11)	−36.20	−20.62	−18.46
6CHZ	−53.89 (CgE18)	−50.84	−19.4	−53.60
3HB5	−98.46 (CgE18)	−15.66	−26.30	−28.50
1U72	−66.88(CgAm11)	−39.67	−22.58	−70.46

### 3.8 Toxicity study

A toxicity study is also important to predict as it influences drug safety and efficacy. Various parameters have been studied, some of which are tabulated in Table 6, which are AMES toxicity and Max. tolerated dose, oral rat acute toxicity, oral rat chronic toxicity, hepatotoxicity, and skin sensitization. It can be observed that all the ligands show no AMES toxicity. The maximum tolerated dose level is from −0.417 mg/kg/day to 0.319 mg/kg/day. This indicates that a maximum of 0.319 mg/kg/day could be administered to patients. Based on the results, oral rat acute toxicity levels range from 1.773 mol/kg to 3.221 mol/kg, and oral rat chronic toxicity levels range from 3.453 mg/kg/day to 4.64 mg/kg/day. Almost all the ligands are free from hepatotoxicity except CgE18, CgAm13, CgE11, and CgTh, and no ligands showed skin sensitization.

**TABLE 6:** Toxicity value prediction.

S. No.	AMES toxicity	Max. tolerated dose (human) mg/kg/day	Oral rat acute toxicity (LD_50_) (mol/kg)	Oral rat chronic toxicity (mg/kg/day)	Hepatotoxicity	Skin sensitization
CgE18	No	0.198	2.515	3.774	Yes	No
CgAm13	No	0.205	2.813	3.453	Yes	No
CgAm11	No	0.245	2.588	4.039	No	No
CgE16	No	−0.417	2.779	4.149	No	No
CgE5	No	0.079	2.685	4.285	No	No
CgE11	No	−0.201	2.731	4.64	Yes	No
CgE9	No	−0.051	2.134	3.559	No	No
CgC	No	−0.245	2.745	4.115	No	No
CgV	No	0.362	2.739	3.932	No	No
CgE	No	0.033	2.855	4.08	No	No
CgTh	No	0.319	2.798	4.123	Yes	No
Epirubicin hydrochloride	No	0.176	2.535	2.305	No	No
5-Fluorouracil	No	1.318	1.773	1.648	No	No
Methotrexate	No	−0.7	3.221	2.836	Yes	No

## 4 Conclusion

The development of novel bioactive compounds for breast cancer treatment is critical to addressing the evolving challenges and complexities of the disease. Chlorogenic acid exhibits promising anticancer potential; however, a detailed mechanistic investigation has not been conducted. Hence, a series of chlorogenic acid derivatives were subjected to computational studies, including molecular docking, ADME, druglikeness, toxicity, and PASS, to design a novel potential inhibitor for breast cancer treatment. SAR analysis was also conducted to enhance the refinement of designed compounds. Highlights of SAR revealed that ester derivatives of CGA exhibited more favorable bindings than anilides, amides, and triazole ring substitutions. The presence of free hydroxyl groups in the CGA molecule is crucial for facilitating hydrogen bonding with essential amino acid residues of the targeted proteins. This analysis enabled us to identify 11 CGA derivatives that possess significant inhibitory activity due to their excellent binding affinities toward breast cancer proteins. Among all the selected derivatives, CgE18, CgE11, CgAm13, CgE16, and CgE9 have astonishing interaction, excellent binding energy, and better stability in the active site of targeted proteins. The outcomes of this study bestow new insights into chlorogenic acid derivatives, and most of them showed excellent binding, even better than the standard drugs, toward the modeled target proteins, which inspired us to further this study in the future.

The docking scores of compound CgE18 were −11.63 kcal/mol, −14.15 kcal/mol, and −12.90 kcal/mol against breast cancer PDB IDs 7KCD, 3HB5, and 1U72, respectively. The docking scores of compound CgE11 were −10.77 kcal/mol and −9.11 kcal/mol against breast cancer PDB IDs 3ERT and 6CHZ, respectively, whereas the docking scores of epirubicin hydrochloride were −3.85 kcal/mol, −6.4 kcal/mol, −8.76 kcal/mol, and −10.5 kcal/mol against PDB IDs 7KCD, 3ERT, 6CHZ, and 3HB5. The docking scores of 5-fluorouracil were found to be −5.25 kcal/mol, −3.43 kcal/mol, −3.73 kcal/mol, and −5.29 kcal/mol, against PDB IDs 7KCD, 3ERT, 6CHZ and 3HB5 respectively and the docking scores of the *Dihydrofolate reductase* (DHFR) inhibitor methotrexate were found to be −13.7 kcal/mol, against PDB ID 1U72 which indicates the designed compounds have a better docking score than studied standard drugs.

The fact that almost all derivatives demonstrated exceptional binding affinities, excellent binding energies, and better stability than standard drugs like epirubicin hydrochloride, 5-fluorouracil, and methotrexate is particularly noteworthy. The direct comparison of binding interaction between the CGA derivatives and standard drugs provides compelling evidence of the potential efficacy of the derivatives as breast cancer inhibitors. The predicted favorable pharmacokinetic features, adequate oral absorption, good metabolic transformation, and absence of toxicity of the newly designed derivatives strongly emphasize the potential of these compounds as candidates for breast cancer treatment. This research presents a hopeful direction for breast cancer treatment by identifying novel CGA derivatives as potential drug candidates. While these findings are encouraging, further synthesis, rigorous testing, clinical trials, and optimization are necessary steps to translate these derivatives into effective and safe anticancer agents for the treatment of breast cancer.

## Data Availability

The original contributions presented in the study are included in the article/Supplementary Material; further inquiries can be directed to the corresponding authors.

## Abbreviations

CGA, chlorogenic acid; MTX, methotrexate; QPlogBB, blood–brain barrier partition coefficient; QPlog HERG, estimated IC_50_ value for HERG K+ channel obstruction; QPlogKhsa, human serum albumin binding; QPlogKp, permeation through skin estimation; QPPCaco, apparent Caco-2 cell permeability estimation in nm/sec; QPPMDCK, apparent Madin–Darby canine kidney (MDCK) cell permeability estimation in nm/sec; Log P, partition coefficient in octanol and water; log S, solubility in aqueous media; % HOA, percent human oral absorption.
