# Supplementary material for: In silico design of novel bioactive molecules to treat breast cancer with chlorogenic acid derivatives: a computational and SAR approach
Source: Front Pharmacol. 2023 Dec 12;14:1266833. doi: 10.3389/fphar.2023.1266833 (PMC10751932; doi:10.3389/fphar.2023.1266833)
Supplement: Supplementary file 1 [file Table4.docx]

***In Silico* Designing of Novel Bioactive Molecules to Treat Breast Cancer with Chlorogenic Acid Derivatives: A Computational and SAR Approach**

**Renu Sehrawat^1^, Priyanka Rathee^2^, Pooja Rathee^3^, Sarita Khatkar^4^, Esra Küpeli Akkol^5*^, Anurag Khatkar^3*^, Eduardo Sobarzo-Sánchez^6,7*^**

^1^ School of Medical & Allied Sciences, K. R. Mangalam University, Gurugram-122103, Haryana, India

^2^ Faculty of Pharmaceutical Sciences, Baba Mastnath University, Rohtak 124021, India

^3^Department of Pharmaceutical Sciences, Maharshi Dayanand University, Rohtak, Haryana, India

^4^ Vaish Institute of Pharmaceutical Education and Research, Rohtak, Haryana, India

^5^ Department of Pharmacognosy, Faculty of Pharmacy, Gazi University, Etiler, 06330, Ankara, Turkey

^6^ Instituto de Investigación y Postgrado, Facultad de Ciencias de la Salud, Universidad Central de Chile, 8330507, Santiago, Chile

^7^ Department of Organic Chemistry, Faculty of Pharmacy, University of Santiago de Compostela, Santiago de Compostela 15782, Spain

***Corresponding Author:**

Prof. Dr. Esra Küpeli Akkol

[esrak@gazi.edu.tr](mailto:esrak@gazi.edu.tr)

Prof. Dr. Eduardo Sobarzo-Sánchez

eduardo.sobarzo@ucentral.cl

Dr. Anurag Khatkar

[anuragpharmacy@gmail.com](mailto:anuragpharmacy@gmail.com); [dranurag.pharma@mdurohtak.ac.in](mailto:dranurag.pharma@mdurohtak.ac.in)

**Table 4** Chlorogenic acid derivatives docked against breast cancer PDB ids (7KCD, 3ERT, 6CHZ, 3HB5, and 1U72) indicating docking score, nature of the interaction, and amino acids involved in interaction in the active site.

| **Compound** | **PDB ID** | **Docking Score**  **(kcal/mol)** | **Nature of interaction** | **Amino acid residues in the active site** |
| --- | --- | --- | --- | --- |
| CgE18 | 7KCD | -11.63 | H-Bond interaction | Glu353, Asn532 |
|  |  |  | Pi-Pi stacking | Phe404 |
|  |  |  | Hydrophobic interaction | Leu525, Trp383, Leu384, Met421, Leu387, Ile424, Met388, Phe425, Phe404, Leu391, Leu428, Met343, Leu346, Val533, Val534, Pro535, Leu539, Ala350, Leu354 |
|  | 3ERT | -9.26 | H-Bond interaction | Asp351, Cys530 |
|  |  |  | Hydrophobic interaction | Leu428, Phe404, Met343, Leu391, Ile424, Leu346, Met421, Ala350, Leu525, Leu354, Leu539, Leu536, Pro 535, Val534, Val533, Cys530 |
|  | 6CHZ | -7.17 | H-Bond interaction | 2 hydrogen bond with Glu353, Arg394, Asp351 |
|  |  |  | Hydrophobic interaction | Phe404, Leu391, Ile424, Leu428, Met388, Met421, Leu387, Met522, Leu384, Leu525, Trp383, Met343, Leu346, Leu349, Ala350 |
|  | 3HB5 | -14.15 | H-Bond interaction | Thr190, Gly92, Ser12, 2 Hydrogen bond with Gly94, 2 Hydrogen bond with Gly186 |
|  |  |  | Pi-Pi stacking | Phe192 |
|  |  |  | Pi-cation | Arg37 |
|  |  |  | Hydrophobic interaction | Tyr155, Val143, Cys185, Pro187, Val188, Phe226, Val188, Phe192, Leu93, Cys10, Ala91, Ile14, Leu16 |
|  | 1U72 | -12.90 | H-Bond interaction | Phe31, Val115, Ala9, Lys55, Glh30 |
|  |  |  | Hydrophobic interaction | Phe34, Phe31, Trp24, Val115, Leu22, Ala9, Val8,Ile7, Tyr121, Ile16, Ile60, Pro61, Leu67 |
| CgAm13 | 7KCD | -10.31 | H-Bond interaction | Asp351, Ser530 |
|  |  |  | Hydrophobic interaction | Leu525, Met528, Val533, Val534, Pro535, Leu539, Leu354, Ala350, Trp383, Leu384, Leu346, Leu387, Met388, Met343, Leu391, Leu428, Phe404, Phe425, Ile424, Met421 |
|  | 3ERT | -7.8 | H-Bond interaction | Asp351, Ala350 |
|  |  |  | Hydrophobic interaction | Leu391, Phe404, Met388, Leu428, Leu387, Met343, Met421, Leu384, Trp383, Leu346, Leu525, Ala350, Leu354, Val533, Val534, Leu536, Leu539 |
|  | 6CHZ | -6.5 | Pi-Pi stacking | Phe404 |
|  |  |  | Hydrophobic interaction | Leu539, leu536, Leu525, Met421, Leu428, Ile424, Phe404, Met343, Leu391, Leu346, Met388, Leu387, Leu349, Ala350, Leu384, Trp383, Leu354 |
|  | 3HB5 | -7.16 | H-Bond interaction | Gly186, Val188, Ile14, Gly15, Ser12, Ser11, Asn90, 2 hydrogen bond with Gly92 |
|  |  |  | Pi-cation | Arg37 |
|  |  |  | Hydrophobic interaction | Tyr155, Cys185, Val188, Phe226, Phe192, Leu16, Ile14, Cys10, Val113, Val66, Leu64, Ala91, Leu93 |
|  | 1U72 | -11.90 | H-Bond interaction | Glh30, Val115 |
|  |  |  | Pi-cation | Arg70 |
|  |  |  | Hydrophobic interaction | Phe34, Phe31, Val115, Ala9, Val8, Ile7, Tyr121, Ile16, Leu22, Trp24, Ile60 |
| CgAm11 | 7KCD | -9.95 | H-Bond interaction | Asn532 |
|  |  |  | Pi-Pi stacking | Phe404 |
|  |  |  | Hydrophobic interaction | Leu525, Val533, Leu539, Pro535, Leu354, Trp383, Leu384, Ala350, Leu349, Leu382, Met388, Leu346, Leu391, Met393, Phe404, Leu428, Phe425, Ile424, Met421 |
|  | 3ERT | -8.5 | H-Bond interaction | Asp351 |
|  |  |  | Hydrophobic interaction | Tyr526, Leu525, Met421, Ile424, Cys530, Val533, Val534, Pro535, Leu536, Leu354, Ala350, Trp383, Leu384, Leu346, Leu387, Met388, Met343, Leu428, Leu391, Phe404, Ile424 |
|  | 6CHZ | -6.5 | Pi-Pi stacking | Phe404 |
|  |  |  | Hydrophobic interaction | Leu428, Ile424, Met421, Leu525, Cys530, Ala350, Leu349, Trp383, Leu384, Leu536, Leu387, Leu536, Leu387, leu346, Met388, Phe404, Met343, Leu391 |
|  | 3HB5 | -11.45 | H-Bond interaction | Gly141, Ser12, Ser11, Gly92, Arg37, Thr140 |
|  |  |  | Hydrophobic interaction | Cys185, Phe192, Ile14, Cys10, Ala91, Leu93, Val113, Val66, Leu64, Leu36 |
|  | 1U72 | -11.89 | H-Bond interaction | Ser118, Thr146, 2 hydrogen bond with Glh30 |
|  |  |  | Pi-stacking | Phe34 |
|  |  |  | Halogen bond | Gly117, Lys55, Thr56 |
|  |  |  | Hydrophobic interaction | Tyr33,Trp24, Leu22, Ile16, Val115, Phe34, Val8, Ala9, Ile7, Val120, Tyr121 |
| CgE16 | 7KCD | -7.27 | H-Bond interaction | Asp351, Ser530 |
|  |  |  | Hydrophobic interaction | Leu525, Met528, Val533, Val534, Pro535, Leu391, Met343, Leu346, Met388, Leu387, Leu346, Phe425, Phe404, Ile424, Leu384, Trp383, Ala350, Leu354, Leu539 |
|  | 3ERT | -9.49 | H-Bond interaction | Asp351 |
|  |  |  | Hydrophobic interaction | Trp383, Leu384, Met421, Leu387, Met388, Ile424, Leu428, Leu391, Met343, Phe404, Leu346, Ala350, Leu354, Met528, Tyr526, Leu525, Met522 |
|  | 6CHZ | -1.5 | H-Bond interaction | 2 hydrogen bond with Glu353, Arg391, Hie524 |
|  |  |  | Hydrophobic interaction | Leu391, Phe404, Met421, Leu428, Met388, Leu387, Ile421, Leu384, Trp383, Leu525, Met522, Met343, Leu346, Leu349, Ala350, Leu354 |
|  | 3HB5 | -10.40 | H-Bond interaction | Val188, Ser12, Val66, Leu64, Thr190 |
|  |  |  | Pi-cation | Arg37 |
|  |  |  | Hydrophobic interaction | Cys185, Pro187, Val188, Phe192, Ile14, Val113, Val66, Leu64, Leu36, Leu93, Ala91, Tyr155, Phe226, Val143 |
|  | 1U72 | -11.83 | H-Bond interaction | Glh30, Ile7, Ala9, Lys55, Thr146 |
|  |  |  | Hydrophobic interaction | Leu62, Phe34, Phe31, Ile7, Trp24, Val8, Ala9, Leu22, Tyr121, Ile16, Val115, Ile60, Pro61 |
| CgE5 | 7KCD | -8.23 | H-Bond interaction | Asp351 |
|  |  |  | Hydrophobic interaction | Leu354, Ala350, Pro535, Leu349, Val533, Leu525, Phe404, Leu346, Met343, Leu428, Leu391, Met388, Leu387, Ile424, Met421, Leu384, Trp383 |
|  | 3ERT | -7.9 | H-Bond interaction | Asp351 |
|  |  |  | Hydrophobic interaction | Met528, Leu525, Leu384, Trp383, Cys530, Val533, Leu536, Ala350, Leu349, Leu346, Phe404, Met343, Leu391, Met388, Leu387 |
|  | 6CHZ | -6.12 | H-Bond interaction | Asp351 |
|  |  |  | Hydrophobic interaction | Ile424, Leu346, Met421, Leu 349, Ala350, Leu354, Leu536, Trp383, Leu525, Leu525, Leu384, Phe404, Leu387, Met388, Leu428, Leu391 |
|  | 3HB5 | -5.35 | H-Bond interaction | Thr190, Asn90, Gly15, Gly141, Lys159, Thr190 |
|  |  |  | Hydrophobic interaction | Tyr155, Cys185, Leu162, Leu16, Ile14, Ala91, Cys10, Leu36, Phe192, Ala191 |
|  | 1U72 | -11.65 | H-Bond interaction | Asp21, Glh30, Thr146, 2 hydrogen bond with Val115 |
|  |  |  | Hydrophobic interaction | Leu22, Trp21, Val115,Phe31, Tyr121, Phe34, Phe31, Ile7, Val8, Ala9, Ile16 |
| CgE11 | 7KCD | -4.3 | H-Bond interaction | Asp351, Asn532, Ser530, Ser341 |
|  |  |  | Hydrophobic interaction | Val418, Met342, Met343, Met528, Val533, Pro535, Leu354, Leu539 |
|  | 3ERT | -10.77 | H-Bond interaction | Asp351 |
|  |  |  | Pi-Pi stacking | Tyr526 |
|  |  |  | Hydrophobic interaction | Met522, Leu525, Met528, Cys530, Val533, Val534, Pro535, Leu536, Leu539, Met421, Ile424, Phe404, Leu428, Met343, Leu391, Met388, Leu346, Ala350, Leu384, Trp383, Leu354 |
|  | 6CHZ | -9.11 | H-Bond interaction | Asp351 |
|  |  |  | Hydrophobic interaction | Met388, Leu387, Met343, Leu428, Phe404,Leu525, Ile424, Tyr526, Met421, Cys530, Val533, Val534, Pro535, Leu536, Leu539, Leu354, Ala354, Leu346, Leu384, Trp383 |
|  | 3HB5 | -12.91 | H-Bond interaction | Gly186, Gly15, Gly92, Ser12, Thr190 |
|  |  |  | Pi-cation | 2 bond with Lys195 |
|  |  |  | Hydrophobic interaction | Tyr155, Val143, Cys185, Pro187, Val188, Phe226, Ala191, Phe192, Val196, Leu93, Ala91, Cys10, Ile14, Leu16 |
|  | 1U72 | -11.44 | H-Bond interaction | Glh30,2 hydrogen bond with Asp21 |
|  |  |  | Pi-Pi stacking | Phe31, Phe34 |
|  |  |  | Salt bridge | Asp21 |
|  |  |  | Hydrophobic interaction | Ile7, Val8, Ala9, Val115, Tyr121, Ile16, Leu22, Trp24, Pro24, Leu67, Phe34, Phe31, Pro61, Ile60 |
| CgE9 | 7KCD | -8.7 | H-Bond interaction | Glu353, Arg 394, 2 hydrogen bond with Asn532, |
|  |  |  | Hydrophobic interaction | Val533, Val534, Pro535, Leu539, Leu354, Ala350, Leu349, Leu391, Phe404, Met388, Leu428, Leu387, Leu346, Met343, Leu525, Trp383, Leu384, Phe425, Ile424, Met421 |
|  | 3ERT | -8.82 | H-Bond interaction | Cys530, 2 hydrogen bond with Asp351 |
|  |  |  | Hydrophobic interaction | Leu354, Ala350, Leu346, Met421, Met343, Phe404, Leu391, Leu428, Met388, Leu387, Leu525, Tyr526, Leu384, Trp383, Met528, Cys530, Val533, Leu536 |
|  | 6CHZ | -7.04 | H-Bond interaction | Glu353, Arg394 |
|  |  |  | Hydrophobic interaction | Trp383, Leu384, Leu387, Met388, Leu391, Ala 350, Leu349, Phe404, Leu346, Met343, Leu525 |
|  | 3HB5 | -12.11 | H-Bond interaction | Thr190, Ser12, Ser11, Cys10, Gly92, Arg37, 2 hydrogen bond Gly 186 |
|  |  |  | Hydrophobic interaction | Phe226, Val188, Pro187, Cys185, Val143, Tyr155, Leu16, Ile14, Cys10, Ala91, Leu93 |
|  | 1U72 | -11.355 | H-Bond interaction | Arg70, Pro66, Gln35, Asn64, Ala9, Val115 |
|  |  |  | Hydrophobic interaction | Leu67, Pro66, Phe34, Phe31, Pro61, Ile60, Phe31, Tyr121, Val115, Ile116, Val8, Ala9, Leu22 |
| CgC | 7KCD | -9.24 | H-Bond interaction | Asp351 |
|  |  |  | Hydrophobic interaction | Pro535, Val534, Val533, Met528, Leu525, Ile424, Leu391, Leu354, Leu428, Met388, Leu387, Ala350, Leu349, Leu384, Phe404, Trp383, Leu539, Leu346, Met343 |
|  | 3ERT | -9.05 | H-Bond interaction | Asp351 |
|  |  |  | Hydrophobic interaction | Leu354, Ala350, Trp383, Leu384, Leu346, Leu387, Met388, Met343, Leu391, Phe404, Leu539, Leu536, Pro535, Val534, Val533, Cys530, Met528, Tyr526, Leu525 |
|  | 6CHZ | -9.35 | H-Bond interaction | Asp 351 |
|  |  |  | Hydrophobic interaction | Met342, Leu346, Leu349, Ala350, Leu354, Met522, Leu536, Leu525, Cys530,Tyr526, Trp383, Leu384, Leu387, Phe401, Met388, Leu39 |
|  | 3HB5 | -9.03 | H-Bond interaction | Thr190, Val66, Leu64, Gly9, Asn90, Ser12, Gly15 |
|  |  |  | Hydrophobic interaction | Tyr155, Cys185, Val188, Pro187, Phe192, Val113, Val66, Leu64, Leu93, Ala91, Cys10, Ile14, Leu16, Phe226 |
|  | 1U72 | -11.355 | H-Bond interaction | Val115, Lys55, Asp21 |
|  |  |  | Pi-Pi stacking | Phe31, Phe34 |
|  |  |  | Hydrophobic interaction | Phe34, Phe31, Ile60, Pro61, Leu67, Ile7, Val8, Ala9, Val115, Ile16, Tyr121, Leu22, Trp24 |
| CgV | 7KCD | -8.15 | H-Bond interaction | Asp351, Ser530 |
|  |  |  | Hydrophobic interaction | Met528, Leu525, Met343, Val533, Val534, Pro535, Leu539, Trp383, Leu384, Ala350, Ile424, Leu387, Met388, Leu428, Phe404, Leu391 |
|  | 3ERT | -8.93 | H-Bond interaction | Cys530 |
|  |  |  | Hydrophobic interaction | Leu525, Met528, Cys530, Val533, Leu536, Leu354, Ala350, Trp383, Leu384, Met421, Phe404, Leu428, Leu387, Ile424, Leu346, Met388, Met343, Leu391 |
|  | 6CHZ | -3.98 | H-Bond interaction | 2 Hydrogen bond with Asp351 |
|  |  |  | Hydrophobic interaction | Leu536,, Val533, Lys530, Met528, Tyr526, Leu525, Met522, leu354, Ala350, Ile425, Leu428, Leu346, Met343, Phe404, Leu391, Met388, Leu387, Leu384, Trp393 |
|  | 3HB5 | -10.40 | H-Bond interaction | Gly94, Asn90, Tyr155, Gly15, Ser12, Gly9 |
|  |  |  | Hydrophobic interaction | Cys185, Pro187, Val188, Phe192, Ala91, Val196, Leu93, Tyr155, Phe226, Leu16, Ile14, Cys10 |
|  | 1U72 | -10.67 | H-Bond interaction | Glh30, Lys55 |
|  |  |  | Pi-Pi stacking | Phe31 |
|  |  |  | Hydrophobic interaction | Leu67, Phe34, Phe31, Ile7, Val8, Ala9, Trp24, Leu22, Ile16, Tyr121, Ile60, Pro61, Val115 |
| CgE | 7KCD | -3.4 | H-Bond interaction | Asp351, Ser530 |
|  |  |  | Hydrophobic interaction | Leu525, Met528, Val533, Val534, Pro535, Leu354, Trp383, Leu384, Met421, Leu387, Ala350, Met388, Ile424, Phe404, Phe425, Leu391, Leu346, Leu428, Met343 |
|  | 3ERT | -8.93 | H-Bond interaction | Asp351 |
|  |  |  | Hydrophobic interaction | Leu354, Ala350, Trp383, Leu384, Leu386, Leu387, Met388, Leu391, Ile424, Leu428, Phe404, Met421, Leu536, Pro535, Val533, Cys530, Met528, Tyr526, Leu525 |
|  | 6CHZ | -3.98 | H-Bond interaction | 2 hydrogen bond with Glu353 |
|  |  |  | Pi-Pi stacking | Phe 404 |
|  |  |  | Hydrophobic interaction | Leu428, Met421, Ile424, Leu384, Trp383, Leu387, Met388, Leu391, Leu354, Leu525, Met 528, Cys530, Met343, Leu346, Leu536, Leu349, Ala350, Leu354 |
|  | 3HB5 | -10.40 | H-Bond interaction | Gly15, Gly9, Glu194, Lys195 |
|  |  |  | Hydrophobic interaction | Phe226, Val143, Ala91, Tyr155, Cys185, Leu16, Ile14, Pro187, Val188, Ala191, Cys10, Phe192 |
|  | 1U72 | -7.79 | H-Bond interaction | Glh30, Phe31 |
|  |  |  | Hydrophobic interaction | Leu67, Phe34, Phe31, Ile7, Val8, Ala9, Trp24, Leu22, Ile16, Val115, Tyr121, Ile60, Pro61 |
| CgTh | 7KCD | -8.83 | H-Bond interaction | Glu353, Asn532 |
|  |  |  | Pi-Pi stacking | Phe 404 |
|  |  |  | Hydrophobic interaction | Met343, Leu346, Leu349, Val533, Val534, Ala350, Pro535, Leu354, Leu391, Leu428, Phe404, Met388, Leu387, Leu384, Trp383, Ile424, Met421 |
|  | 3ERT | -8.25 | H-Bond interaction | Asp351, Glu353 |
|  |  |  | Hydrophobic interaction | Ile424, Met421, Leu525, Leu536, Leu354, Ala350, Leu349, Trp383, Leu384, Leu387, Met388, Phe404, Leu391, Leu346, Met343 |
|  | 6CHZ | -2.17 | H-Bond interaction | 2 hydrogen bond with Glu353, Arg394, Hie524 |
|  |  |  | Pi-Pi stacking | Phe 404 |
|  |  |  | Hydrophobic interaction | Leu391, Phe404, Met388, Leu387, Met421, Leu428, Ile424, Leu384, Trp383, Leu525, Met343, Leu346, Leu349, Ala 350 |
|  | 3HB5 | -9.4 | H-Bond interaction | Gly141, Val66, Leu64, Ser12, Thr190 |
|  |  |  | Pi-cation | Arg37 |
|  |  |  | Hydrophobic interaction | Tyr155, Cys185, Ala91, Leu36, Leu93, Val113, Val66, Leu64, Ile14, Phe192 |
|  | 1U72 | -7.79 | H-Bond interaction | Asn64, Ala9 |
|  |  |  | Salt Bridge | Lys55 |
|  |  |  | Hydrophobic interaction | Leu67, Pro61, Ile60, Val115, Ile16, Phe31, Phe34, Ile7, Val8, Ala9, Tyr121, Trp24, Pro23, Leu22, Ile16 |
| Chlorogenic acid | 7KCD | -5.26 | H-Bond interaction | Asn532 |
|  |  |  | Hydrophobic interaction | Leu525, Leu346, Met421, Met342, Ile424, Phe425, Leu428, Leu391, Phe404, Met388, Leu387, Leu384, Trp383, Pro535, Leu354, Val534, Val533 |
|  | 3ERT | -7.9 | H-Bond interaction | 2 H-bond Asp351 |
|  |  |  | Hydrophobic interaction | Trp383, Leu525, Leu384, Leu387, Leu391, Phe404, Met343, Ile424, Leu346, Met421, Ala350, Leu354, Leu536 |
|  | 6CHZ | -6.11 | H-Bond interaction | Cys530, 2 H- bond with Asp351, |
|  |  |  | Hydrophobic interaction | Leu525, Met522, Met388, Leu387, Leu384, Trp383, Cys530, Leu346, Phe404, Leu536, Leu349, Ala350. |
|  | 3HB5 | -10.22 | H-Bond interaction | Hie221, Ser142, 2 H-bond Glu282 |
|  |  |  | Hydrophobic interaction | Leu262, Phe259, Val225, Phe259, Val225, Phe226, Val143, Cys185, Pro187, Val188, Tyr155, Met193, Phe192, Leu149, Met279, Tyr218, Val283 |
|  | 1U72 | -10.2 | H-Bond interaction | Lys55, Ala9, 2 H-bond with Val115, 2 H-bond with Thr146 |
|  |  |  | Hydrophobic interaction | Ile16, Tyr121, Val115, Ile7, Val8, Ala9, Phe31, Phe34, Trp24, Leu22 |
| Methotrexate | 1U72 | -13.7 | H-Bond interaction | 2 H-bond with Glh 30, Val 115, Ile 7, 2 H-bond with Asn 64 |
|  |  |  | Salt Bridge | Gln 35, Arg 70 |
|  |  |  | Hydrophobic interaction | Tyr 121, Val 115, Ile 7,Val 8, Ala 9, Phe 31, Phe 34, Tyr 33, Ile 60, Pro 61, Leu 67 |
| Epirubicin hydrochloride | 7KCD | -3.85 | H-Bond interaction | Asn532 |
|  |  |  | Hydrophobic interaction | Val533, Val534, Pro535, Trp383, Leu539, Met543, Val355, Leu354 |
|  | 3ERT | -6.4 | H-Bond interaction | Asp351, Leu525 |
|  |  |  | Hydrophobic interaction | Leu354, Ala350, Trp383, Leu536, Val533, Cys530, Met528, Tyr526, Leu525, Met522 |
|  | 6CHZ | -8.76 | H-Bond interaction | Leu536, Glu 380, Tyr526, Cys530 |
|  |  |  | Hydrophobic interaction | Leu536, Pro535, Val533, Cys530, Met528, Tyr526, Leu525, Met 522, Ala350, Leu354, Trp 383 |
|  | 3HB5 | -10.5 | H-Bond interaction | Thr140, Lys159, Gly92, Asn90, 2 hydrogen bond with Thr190 |
|  |  |  | Hydrophobic interaction | Phe226, Tyr155, Val143, Leu162, Ala91, Phe226, Cys185, Pro187, Val188, Ala91, Ile14, Phe192, Met93 |
|  | 1U72 | --- | ---- | ---- |
| 5-Fluorouracil | 7KCD | -5.25 | H-Bond interaction | Glu353, Arg394 |
|  |  |  | Pi-Pi stacking | Phe404 |
|  |  |  | Hydrophobic interaction | Leu346, Leu349, Ala350, Phe404, Leu391, Leu428, Met388, Leu387 |
|  | 3ERT | -3.43 | H-Bond interaction | Glu353, Arg394 |
|  |  |  | Hydrophobic interaction | Leu384, Leu387, Met388, Leu391, Ala350, Leu349, Leu346 |
|  | 6CHZ | -3.73 | H-Bond interaction | Glu353 |
|  |  |  | Hydrophobic interaction | Leu387, Met388, Leu384, Leu346, Leu349, Ala350, Phe404, Leu391 |
|  | 3HB5 | -5.29 | H-Bond interaction | Gly9, Asn90, Gly15, 2 hydrogen bonding with Gly92 |
|  |  |  | Hydrophobic interaction |  |
|  |  |  |  |  |
